# Aliphatic Polyester Recognition and Reactivity at the Active Cleft of a Fungal Cutinase

**DOI:** 10.1021/acs.jcim.5c00739

**Published:** 2025-04-24

**Authors:** Pietro Vidossich, Madushanka Manathunga, Andreas W. Götz, Kenneth M. Merz, Marco De Vivo

**Affiliations:** † Laboratory of Molecular Modeling and Drug Discovery, Istituto Italiano di Tecnologia, Via Morego 30, 16163 Genoa, Italy; ‡ Department of Chemistry and Department of Biochemistry and Molecular Biology, Michigan State University, 578 S. Shaw Lane, East Lansing, Michigan 48824-1322, United States; § San Diego Supercomputer Center, University of California, San Diego, 9500 Gilman Drive, La Jolla, California 92093-0505, United States

## Abstract

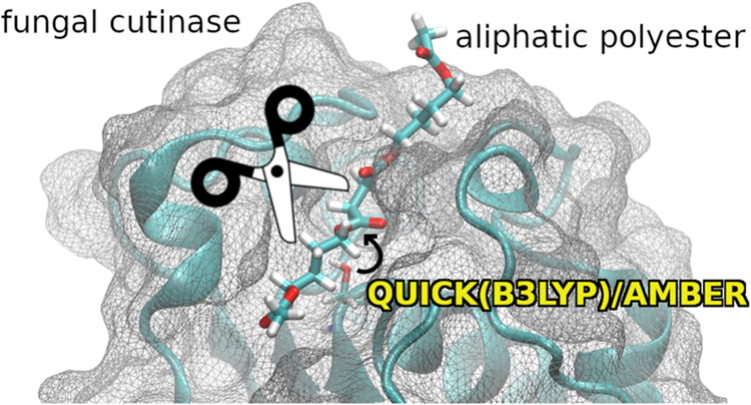

Protein engineering of cutinases is a promising strategy for the biocatalytic degradation of non-natural polyesters. We report a mechanistic study addressing the hydrolysis of the aliphatic polyester poly(butylene succinate, or PBS) by the fungal *Apergillus oryzae* cutinase enzyme. Through atomistic molecular dynamics simulations and advanced alchemical transformations, we reveal how three units of a model PBS substrate fit the active site cleft of the enzyme, interacting with hydrophobic side chains. The substrate ester moiety approaches the Asp–His–Ser catalytic triad, displaying catalytically competent conformations. Acylation and deacylation hydrolytic reactions were modeled according to a canonical esterase mechanism using umbrella sampling simulations at the quantum mechanical/molecular mechanical DFT(B3LYP)/6–31G**/AMBERff level. The free energy profiles of both steps show a high-energy tetrahedral intermediate resulting from the nucleophilic attack on the ester’s carboxylic carbon. The free energy barrier of the acylation step is higher (20.2 ± 0.6 kcal mol^–1^) than that of the deacylation step (13.6 ± 0.6 kcal mol^–1^). This is likely due to the interaction of the ester’s carboxylic oxygen with the oxyanion hole in the reactive conformation of the deacylation step. In contrast, these interactions form as the reaction proceeds during the acylation step. The formation of an additional hydrogen bond interaction with the side chain of Ser48 is crucial to stabilizing the developing charge at the carboxylic oxygen, thus lowering the activation free energy barrier. These mechanistic insights will inform the design of enzyme variants with improved activity for plastic degradation.

## Introduction

The management of disposable plastic waste has become a global environmental challenge.^1^ Most waste ends up buried in landfills or released into the ecosystem.^2^ Only a minor part of such waste is recycled. Further aggravating it, recent studies report that even the correct implementation of the current policies for plastic waste management will not be effective in reverting the accumulation of waste in the environment.^3^ This worrying scenario calls for the immediate development of new, environmentally friendly technologies for a circular plastic economy.^4^ In this respect, advances have been pursued from different perspectives. On one side, materials research is pursuing the development of innovative polymeric materials that are easier to degrade than commercial plastics. From another perspective, the research attempts to develop cost-effective degrading technologies capable of breaking current polymers into their constituent building blocks, thus enabling a circular plastic economy.

In this context, biotechnological approaches constitute an attractive strategy for plastic degradation compared to chemical and physical treatment.^5^ This approach, put forward more than 50 years ago,^6,7^ has gained considerable attention since discovering the bacterium *Ideonella sakaiensis*.^8^ This bacterium can live on poly(ethylene terephthalate) (PET), used as a carbon source. The activity of *I. sakaiensis* is due to the action of two enzymes, named PETase and MEHase, both belonging to the esterase family of enzymes.^9^ Indeed, the ester bonds that join the building blocks of such polyesters are amenable to hydrolysis by carboxylic ester hydrolases.^10^ Unfortunately, the activity of wild-type PETase and MEHase enzymes toward polyesters is too low for their practical applications. This prompted numerous studies to improve polymer degradation by the protein engineering of more efficacious hydrolytic enzymes.^11^ In particular, efforts focus on the hydrolysis of PET by modified bacterial cutinases, a subfamily of α/β hydrolases responsible for the hydrolysis of cutin, the structural component of higher plant cuticle.^12^ Interestingly, protein engineering efforts were often based on a rational approach, leveraging the available structural data and aided by molecular modeling and machine learning.^13−19^ Moreover, detailed atomistic studies of the reaction mechanism of PET hydrolysis by PETase have been conducted.^20−24^

**Figure 1:**
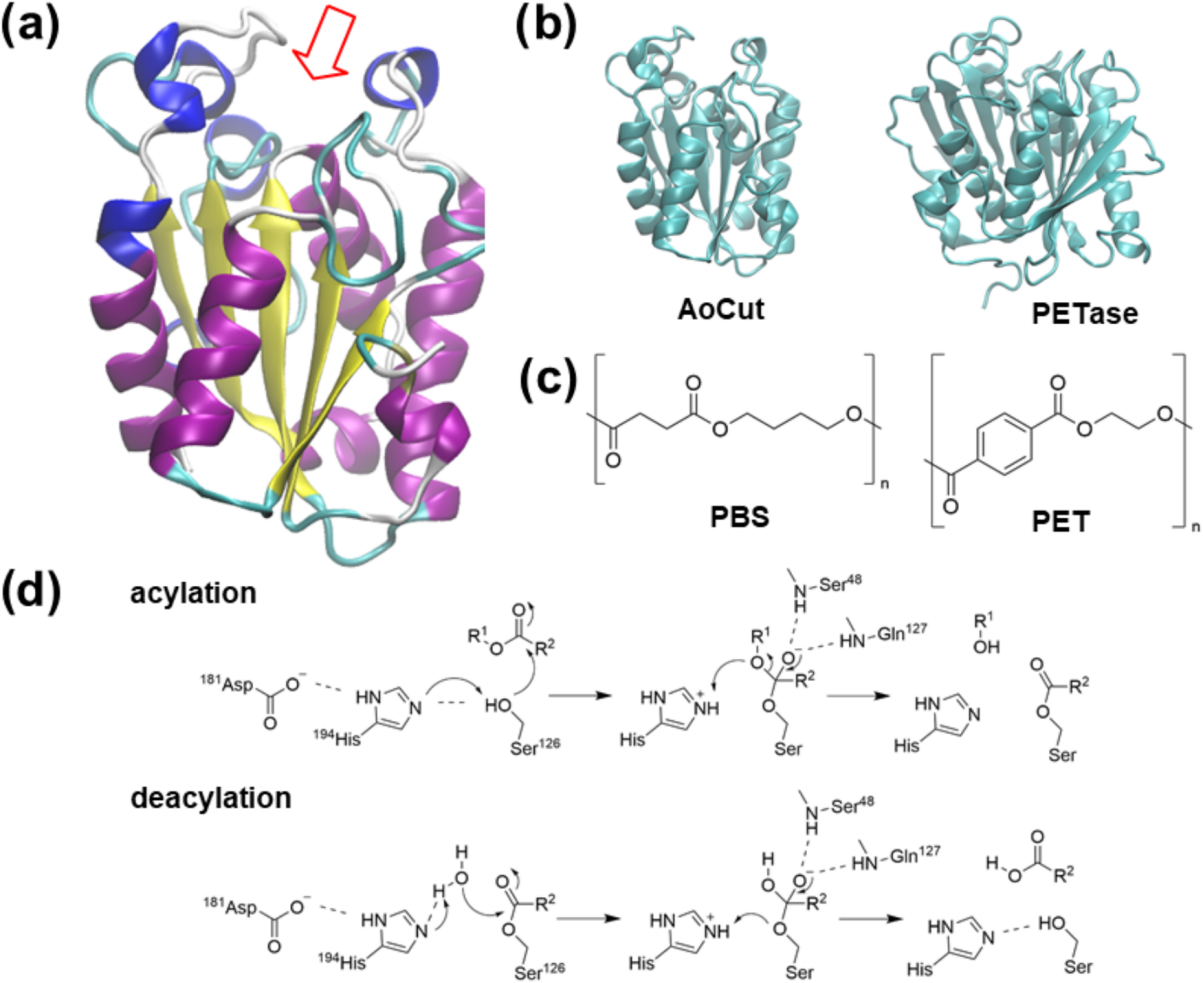
(a) Cutinase from *A. oryzae* (PDB ID: 3GBS); the red arrow points to the active site cleft; (b) *Ao*Cut and PETase share 16% sequence identity, and the common substructures display a RMSD of 3.2 Å; (c) chemical drawings of polyesters PBS and PET; (d) acylation and deacylation steps of the canonical esterase mechanism of ester hydrolysis.

Oddly, less attention has been paid to fungal cutinases and polyesters other than PET. Fungal cutinases share the α/β hydrolases fold with their bacterial counterparts, being smaller and displaying low sequence identity with bacterial cutinases.^25^ Previous studies experimentally investigated the biodegradation activity of the cutinase from *Aspergillus oryzea* (*Ao*Cut, Figure 1a,b), a filamentous fungus used in the food industry for fermentation processes.^26^ AoCut is ∼200 residues long and features the classical elements of the α/β hydrolases fold,^27^ including a central β-sheet comprising five parallel strands surrounded by ten α-helices. Here, catalysis is carried out by a triad of residues, namely, Ser126, Asp181, and His194, with the nucleophilic hydroxyl of Ser126 activated by the flanking His194, in turn assisted by Asp181 (Figure 1d). The oxyanion hole, formed by the backbone amide −NH– groups of Ser48 and Gln127, completes the canonical serine esterase active site.^28^ Furthermore, three disulfide bonds (Cys63-Cys76, Cys37-Cys115, and Cys177-Cys184) are present along the chain, contributing to fold stabilization.

*Ao*Cut has been reported to hydrolyze poly(butylene succinate) (PBS) and poly(butylene succinate-*co*-adipate) (PBSA) in emulsion preparations and films.^29^ Partial hydrolysis was observed for poly(lactic acid) (PLA). Indeed, the biodegradability of aliphatic polyesters, such as PBS, PBSA, and PLA, is higher than that of aromatic polyesters, such as PET. Nevertheless, cost-effective biodegradable recycling systems still need to be developed. Given that the substrate specificity of carboxylic ester hydrolases depends on substrate recognition at the active site cleft, it is thus of great interest to gather mechanistic insight into the biodegradation process of aliphatic polyesters by fungal cutinases, which are characterized by a different cleft architecture compared to bacterial cutinases (Figure 1b).

Here, we report in atomic detail the hydrolytic mechanism of PBS by enzyme *Ao*Cut. Specifically, classical molecular dynamics simulations were used to investigate the recognition of a PBS-like oligomer at the enzymatic active site cleft, followed by hybrid quantum mechanical/molecular mechanical (QM/MM) simulations at the B3LYP/6–31G(d,p)//AMBER level of theory. Importantly, our QM/MM includes full long-range electrostatics in the simulations (through a QM/MM PME approach).^30,31^ In particular, the inclusion of this term correctly handles the long-range electrostatics of charge-separated species resulting from the heterolytic cleavage of bonds, as in the present case. Finally, we could reconstruct the free energy profile for the canonical esterase mechanism of both acylation and deacylation steps, which lead to ester hydrolysis. Together, these findings provide guiding principles for designing enzyme variants with improved activity for polyester degradation.

## Methods

### Model Systems

**Figure 2:**
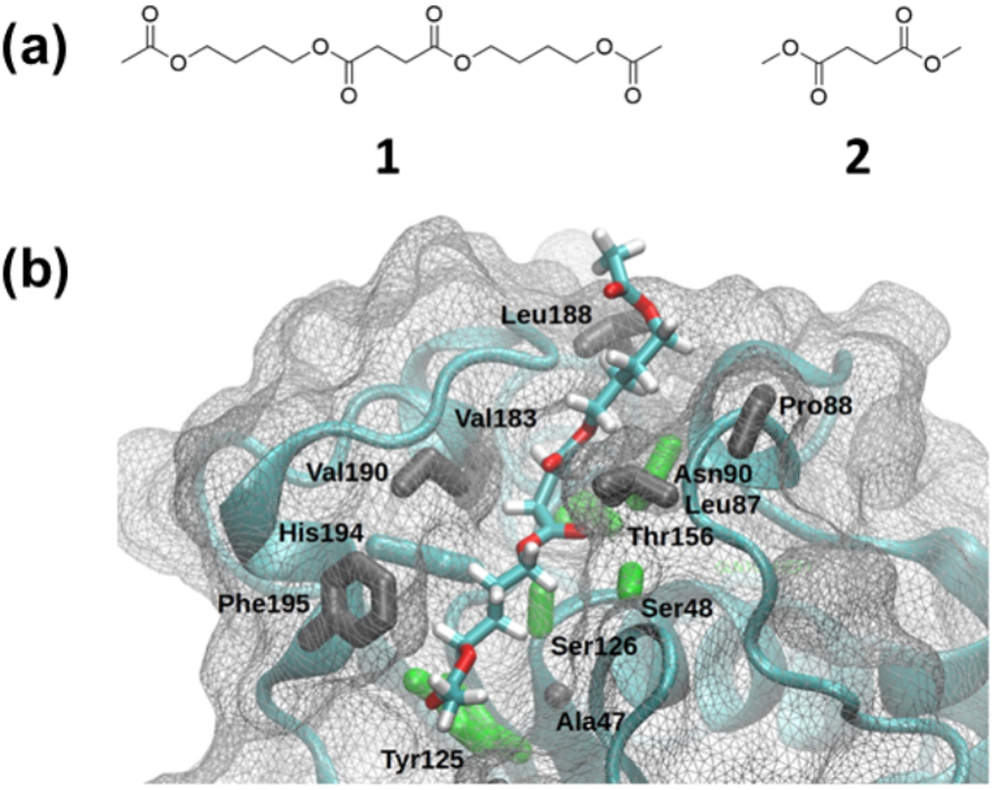
(a) PBS-like oligomer used as model substrate (compound **1**) and dimethyl succinate (compound **2**); (b) *Ao*Cut/**1** Michaelis complex. The amino acid side chains in contact with compound **1** are shown as bold sticks (gray hydrophobic, green polar). The enzyme backbone is rendered as a cyan cartoon, while the enzyme surface as a gray wireframe.

The experimental structure of the apo *Ao*Cut (PDB code 3GBS,^26^ 1.75 Å resolution) was used to build models of the apo enzyme and complexes with substrates **1** and **2** (Figure 2a). The experimental structure included residues 26 to 212. The N- and C-termini were capped with acetyl and *N*-methyl groups, respectively. Protonation states were assigned with PROPKA3, assuming pH 7.^32^ His194 was considered neutral and protonated at the δ nitrogen. The pose of compound **2** in the active site cleft of AoCut was generated *via* docking (described below), while compound **1** was grown from compound **2***via* an alchemical approach (described below). Systems were solvated in simulation boxes extending at least 14 Å from the protein surface. Sodium and chloride ions were added randomly to neutralize the system and reach a final concentration of ∼150 mM. Final models included ∼45,000 atoms in a 80 × 80 × 70 Å^3^ box.

### Classical Molecular Dynamics

Molecular dynamics (MD) simulations were performed with the pmemd module of Amber22.^33,34^ The AMBER-ff14SB force field was used for the protein,^35^ the TIP3P model for water,^36^ and Joung–Chetham parameters for monovalent ions.^37^ GAFF2 bonded and van der Waals parameters were used for compounds **1** and **2**.^38^ RESP partial charges were developed for compounds **1** and **2** and for the acylated form of Ser126 as described in the Supporting Information.^39^ Simulations were performed using a cutoff of 10 Å. Long-range electrostatics were treated with the particle mesh Ewald method.^40^ Bonds involving hydrogen atoms were constrained, allowing a time step of 2 fs.^41^ After solvent equilibration, systems were energy minimized and gently heated while restraining protein backbone atoms to maintain the experimental structure. The Andersen-like temperature-coupling scheme and a Monte Carlo barostat were used to maintain the temperature and pressure close to room-temperature conditions (300 K and 1 bar). About 1 μs MD simulations in the NPT ensemble were performed for the apo AoCut, the AoCut:**2** complex, and the acyl-enzyme intermediate (with and without the R^1^O_But_H product), while 2.5 μs was accumulated for the AoCut:**1** complex.

### Alchemical Molecular Dynamics

The alchemical enhanced sampling (ACES) method was used to “grow” compound **1** from compound **2**.^42^ Note that the objective of the alchemical transformation was to couple compound **1** to the active cleft of *Ao*Cut and not to estimate their affinity. In ACES, intramolecular torsional and electrostatic interactions of the alchemical fragments are scaled with the alchemical parameter λ, thus creating an enhanced sampling decoupled state, which is propagated through the λ range *via* exchange with neighboring replicas according to the replica-exchange algorithm.^43^ By this means, the conformational sampling of compound **1** is increased, facilitating its coupling with the fully flexible protein active site cleft. Specifically, 31 λ windows were employed, each sampled for 5 ns by using a time step of 1 fs, with the λ values manually adjusted to guarantee a 20–30% replica-exchange acceptance rate between neighboring windows. A swap of configurations was attempted every 2 ps. Initial configurations were generated from 1 ns-long alchemical simulations at each λ value, sequentially increasing λ from 0 (corresponding to compound **2**) to 1 (compound **1**). Atoms unique to each compound were treated with smoothstep softcore potentials.^44^ Ten ACES transformations were performed, starting from different initial configurations. Due to the low residence time of compound **2** in the active cleft of *Ao*Cut, we applied a soft harmonic restraint on the H-bonding distances between the carbonyl oxygen of compound **1** and the protein backbone hydrogens of the oxyanion hole. After each transformation, 250 ns unbiased MD simulations were performed for a cumulative simulation time of 2.5 μs.

### QM/MM Molecular Dynamics

Hybrid quantum mechanical/molecular mechanical (QM/MM) simulations were performed with the QUICK code interfaced with the MD engine SANDER available from AmberTools23.^30,45^ Simulations were performed for the complex AoCut:**1**. The QM region included the side chains of the catalytic triad Ser126, His194, and Asp181, the peptide units of the oxyanion hole Ser126-Gln127 and Ala47-Ser48, the side chain of Ser48, and part of compound **1** (the central hydrolyzable unit), accounting for about 80 atoms and a total charge of −1. QM atoms were described by the hybrid B3LYP DFT functional and basis set 6–31G(d,p).^46−49^ The remaining atoms were treated at the MM level, as described above. The QM/MM simulations were performed at constant volume and temperature (300 K) using a time step of 0.5 fs to integrate the equations of motion. The electrostatic cutoff was set to 10 Å. The SCF convergence criterion required a maximum RMS of 5 × 10^–7^ on the change of the density matrix. QM/MM simulations were started from equilibrated configurations from classical MD simulations.

### Umbrella Sampling

**Figure 3:**
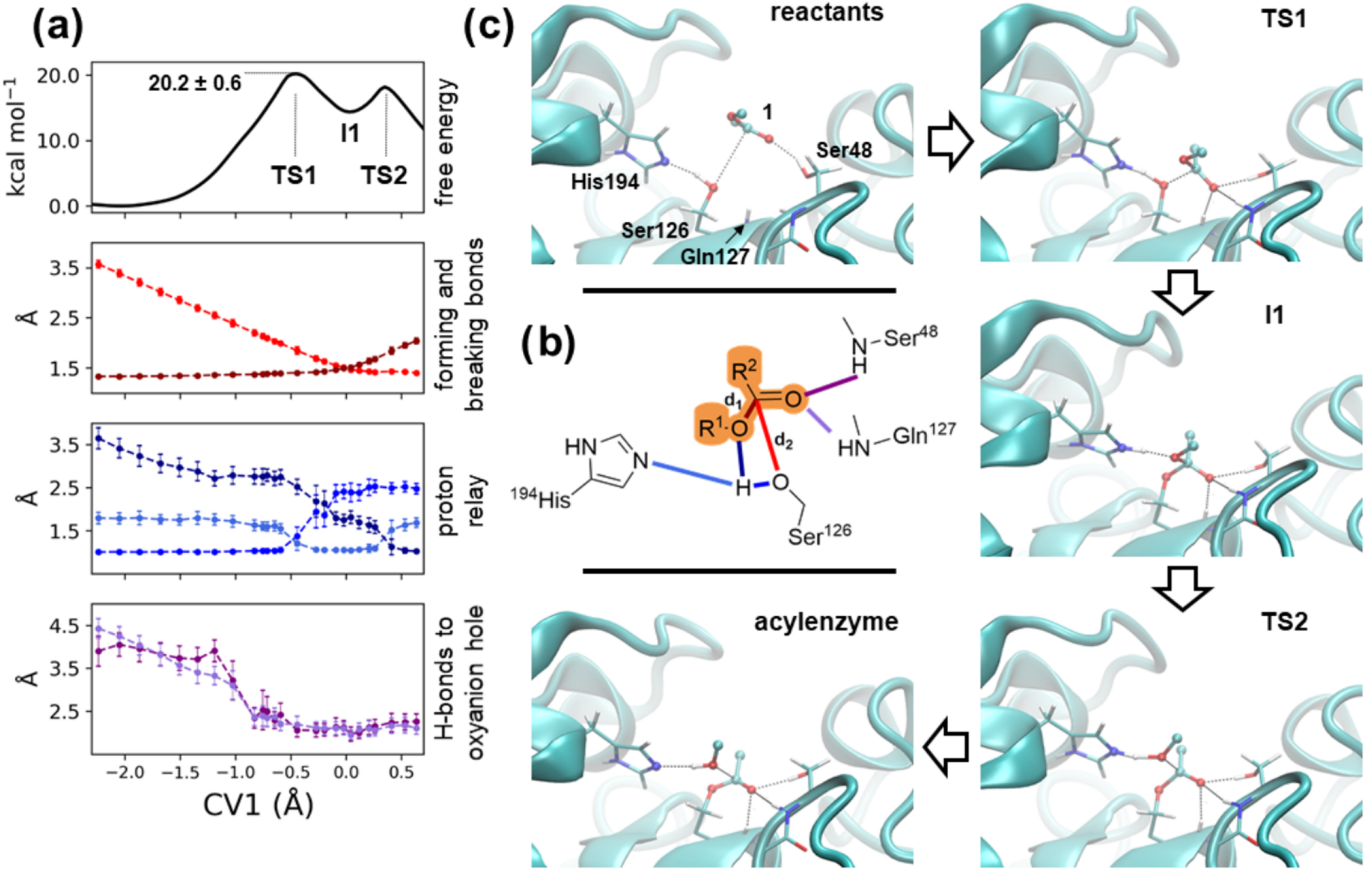
Acylation reaction step. (a) Free energy profile and relevant distances along the collective variable CV1 = *d*_1_ – *d*_2_; (b) chemical drawing highlighting the distances shown in panel (a); (c) representative configurations of reactants, transition states, and intermediate and acyl-enzyme state*s*, labeled according to their corresponding CV1 values. For the sake of clarity, only the hydrolyzable moiety of compound **1** is drawn.

Umbrella sampling (US) QM/MM simulations were performed to reconstruct the potential of mean force of the acylation and deacylation steps (Figure 1d) of the hydrolysis of substrate **1**.^50,51^ The progress of the reaction was controlled by collective variables (CV) defined by the difference of the distances of the breaking and forming bonds (CV1 = *d*_1_ – *d*_2_ in Figure 3 and CV2 = *d*_3_ – *d*_4_ in Figure 5). The CVs range from about −2 (reactants state) to beyond the transition state region (∼0.6) was sampled using about 20 windows, with the value of the CVs restrained at a specific value by a harmonic potential (see Tables S1–S3 for further details on the restraints reference positions and force constants). Initial structures were taken from preliminary steered MD simulations, which employed the same CVs to move the system from reactant to product states. 8 ps simulations were simulated for each window, of which the last 6 ps was used for data analysis. The potential of mean force was reconstructed using umbrella integration.^52^

### Docking

Molecular docking was performed using the Glide program from the Schrodinger suite.^53^ Compound **2** was docked to the active site cleft using a 20 Å side cubic search box centered on the side chain of Ser126. An ensemble docking approach was adopted using 1000 structures randomly extracted from a 1 μs long equilibrium MD simulation of the apo *Ao*Cut. Docking was performed using the Extra Precision (XP) scoring function.^54^

## Results

### Formation of the Cutinase/PBS Michaelis Complex

The first step of the enzymatic reaction is the recognition of the substrate at the active site cleft of the fungal *A. oryzae* cutinase enzyme (*Ao*Cut). Here, we used compound **1** as a model substrate (Figure 2a). We modeled compound **1** in situ, based on the binding of the smaller dimethyl succinate (compound **2** in Figure 2a), which comprises the hydrolyzable unit of PBS. Specifically, we used the alchemical enhanced sampling (ACES) method to “grow” compound **1** from compound **2**, followed by standard MD for a cumulative simulation time of 2.5 μs, which were analyzed to reveal the binding modes of compound **1** in the active cleft of *Ao*Cut (see the Supporting Information for more details).

**Scheme 1:**
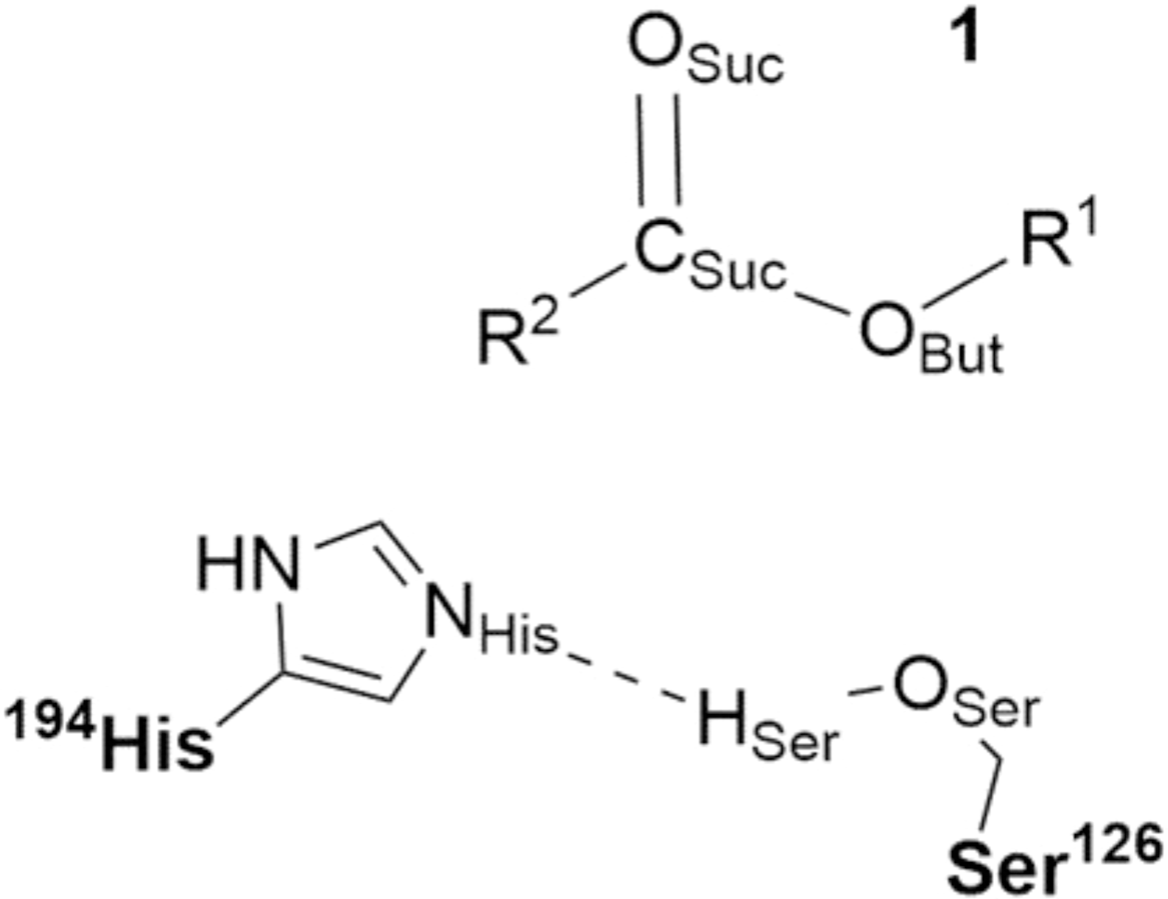
Atom Labels Used to Describe the Acylation Step of the Hydrolysis of Compound **1**, a Poly(butylene succinate)-like Oligomer (Figures 1 and 2)

First, we noted that conformations displaying a distance of <4 Å between the hydrolyzable carbonyl carbon (C_Suc_ in Scheme 1) of compound **1** and the nucleophilic hydroxyl group of Ser126 (O_Ser_ in Scheme 1) accounted for 64% of the cumulative trajectory. Clustering of these frames based on the RMSD of the butanediol–succinate–butanediol moiety of compound **1** showed that the substrate had a recurrent (16%) binding mode in the active site cleft of *Ao*Cut. In these poses, O_Suc_ was *not* inserted in the oxyanion hole. The butanediol–succinate–butanediol moiety was accommodated into a binding canyon of predominantly hydrophobic side chains (Figure 2b). However, polar residues, including the catalytic ones, surrounded the hydrolyzable ester moiety. Indeed, the side chains of Ser48 and Asn90 H-bonded the carboxylic oxygen during the simulation (16 and 29%, respectively).

### Step 1, the Acylation Reaction Mechanism Is Aided by Precise H-Bond Interactions

As noted above, the most recurrent binding mode of substrate **1** in the active site cleft of *Ao*Cut did not show the insertion of the ester into the oxyanion hole. Yet, nearby residues, such as Ser48 and Asn90, could establish H-bonds to the carboxylic oxygen. We explored the reaction mechanism and reconstructed the associated free energy profile by means of umbrella sampling QM/MM simulations, starting from two different configurations, named 1_A and 1_B hereafter, belonging to the most recurrent binding mode of the butanediol–succinate–butanediol moiety of compound 1 in the active site cleft of *Ao*Cut. These two configurations displayed different H-bonding patterns with the ester moiety of the substrate. QM/MM simulations of the reactant states confirmed that both poses were stable, with C_Suc_ remaining close to the nucleophilic side chain oxygen of Ser126. Notably, the free energy profile of **1_A** showed a barrier (20.2 ± 0.6 kcal mol^–1^) that was smaller than that of **1_B** (24.5 ± 0.7 kcal mol^–1^). This result can likely be ascribed to the number of H-bonds formed at the transition state by O_Suc_. We observed three H-bonds for **1_A**, while we observed only two for **1_B**. In the following, we describe the reaction mechanism of the lower energy profile starting from **1_A** (see Figure 3), while that from **1_B** is described in the Supporting Information (Section 6).

For **1_A**, the reaction proceeds with the nucleophilic oxygen of Ser126 (O_Ser_ in Scheme 1) approaching the carboxylic carbon C_Suc_ of the ester moiety (Figure 3c). The free energy profile displays an intermediate (**I1**) at CV1 = 0.0 Å, that is, when the forming and breaking bonds are of equal length (∼1.5 Å, Figure 3a). This state corresponds to a shallow minimum on the free energy profile. The first transition state (**TS1**) is found at CV1 = ∼−0.5 Å, corresponding to a still formed C_Suc_–O_But_ bond (∼1.4 Å, Figure 3), while O_Ser_ is ∼1.8 Å from C_Suc_. At this stage, the transfer of a proton from Ser126 to His194 takes place (Figure 3a,c). The second transition state (**TS2**) is located at CV1 = ∼0.3 Å, corresponding to the breaking of the C_Suc_–O_But_ bond (∼1.8 Å). This occurs when His194, after a change in H-bonding partner, delivers a proton to the leaving O_But_ (Figure 3a,c). Notably, **TS1** is higher in free energy by 2.0 kcal mol^–1^ (Figure 3a) than **TS2**. Also, a pattern of H-bond interaction at the oxyanion hole was formed as the reaction proceeded for CV1 > −0.9 Å (Figure 3a). However, the reactant state did not display such H-bonding interactions. At both transition states, the backbone −NH– of Ser48 and Gln127 interacted with O_Suc_ and, in addition, the latter H-bonded the side chain hydroxyl of Ser48 (Figure 3c). Thus, the O_Suc_ of the ester moiety forms three H-bonds at both **TS1** and **TS2**. At CV1 > 0.55 Å, we verified that the systems were committed to the product states by releasing the restraints and observing the evolution of the systems, which indeed formed the expected acyl-enzyme adduct and the newly created alcohol molecule still bound to the enzymatic cleft (Figure S8).

### Dynamics of the Acyl-enzyme Adduct at the Active Site

**Figure 4:**
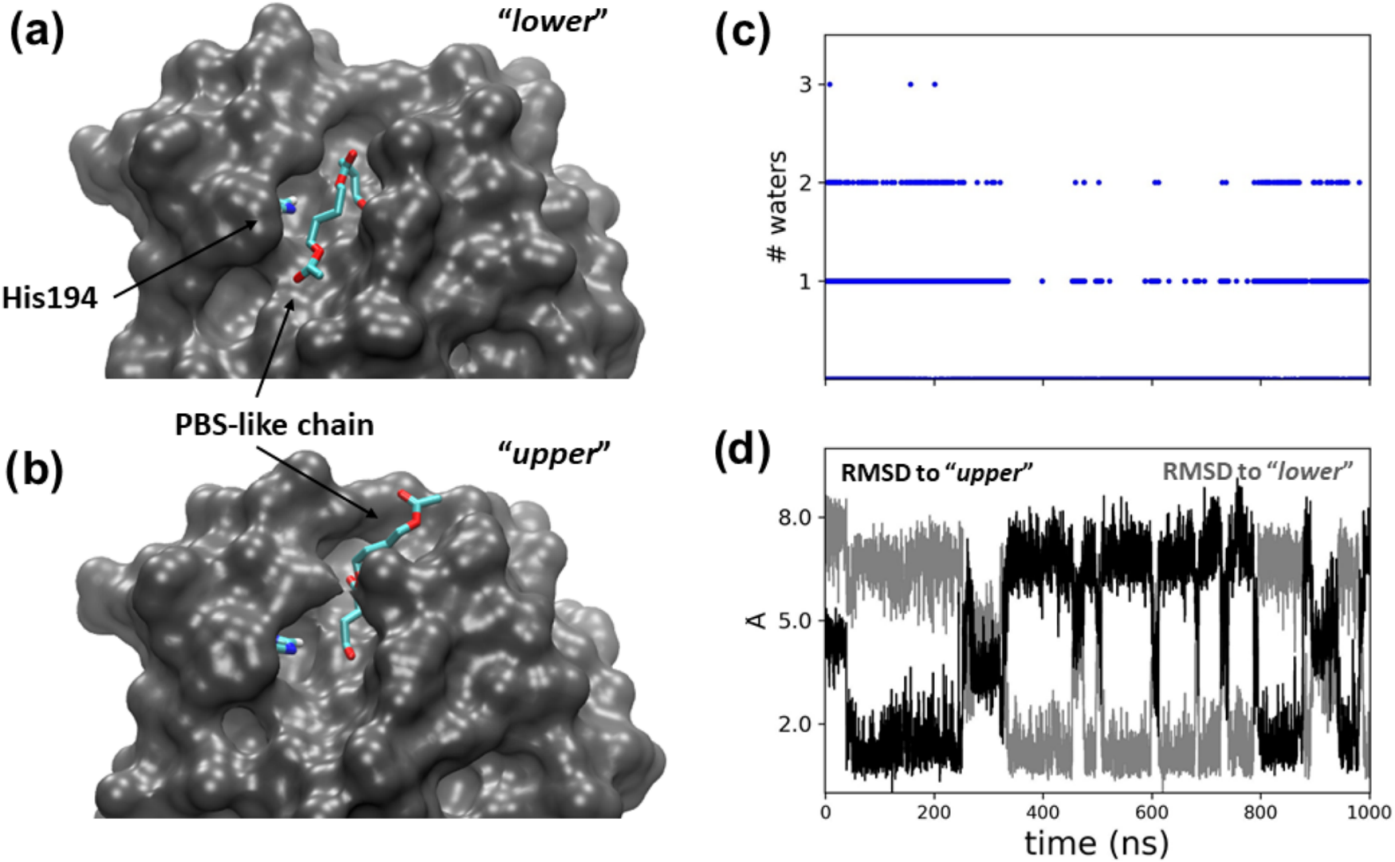
Acyl-enzyme intermediate. (a, b) Binding modes of the PBS-like chain in the active site cleft of *Ao*Cut; (c) the number of water molecule within 3.5 Å from the side chain of His194; (d) RMSD with respect to the two configurations shown in (a) (gray line) and (b) (black line).

The acylation reaction produced an acyl-enzyme adduct, in which C_Suc_ is covalently bound to Ser126, while the newly created alcohol R^1^O_But_H is still bound at the active cleft (Figure 3c). Classical MD simulations of this state showed that R^1^O_But_H can promptly leave the active site cleft and only occasionally (during the 1 μs long simulation) bound back with the alcoholic terminus close to the side chain of Ser126 (Figure S13). To further characterize the conformational dynamics of the acyl-enzyme intermediate alone, we removed the R^1^O_But_H molecule from the system and performed further 1 μs equilibrium MD simulations. This revealed two aspects of how exactly the covalently bound PBS-like fragment sits at the active site cleft of the enzyme. First, O_Suc_ forms H-bond interactions with the oxyanion hole more easily than does compound **1** (Figure S14). This seems primarily due to the spatial proximity imposed by the covalent bond formed by the substrate with Ser126. Second, the substrate chain adopts distinct poses in the active site cleft, occupying either the “*upper*” side or the “*lower*” side of the cleft (Figure 4a,b). This occurrence has relevance to the progress of the reaction, given that water is excluded from approaching the carboxylic carbon from the side of His194 when the PBS-like chain occupies the same side (*i.e.*, the “*lower*” side) of the cleft as that of His194 (Figure 4c). However, the conformational rearrangement appears to take place on the hundreds of nanoseconds time scale, and thus, it is not expected to impact the catalytic rate (Figure 4d).

### Step 2, the Deacylation Reaction Mechanism for Final Product Formation and Release

**Figure 5:**
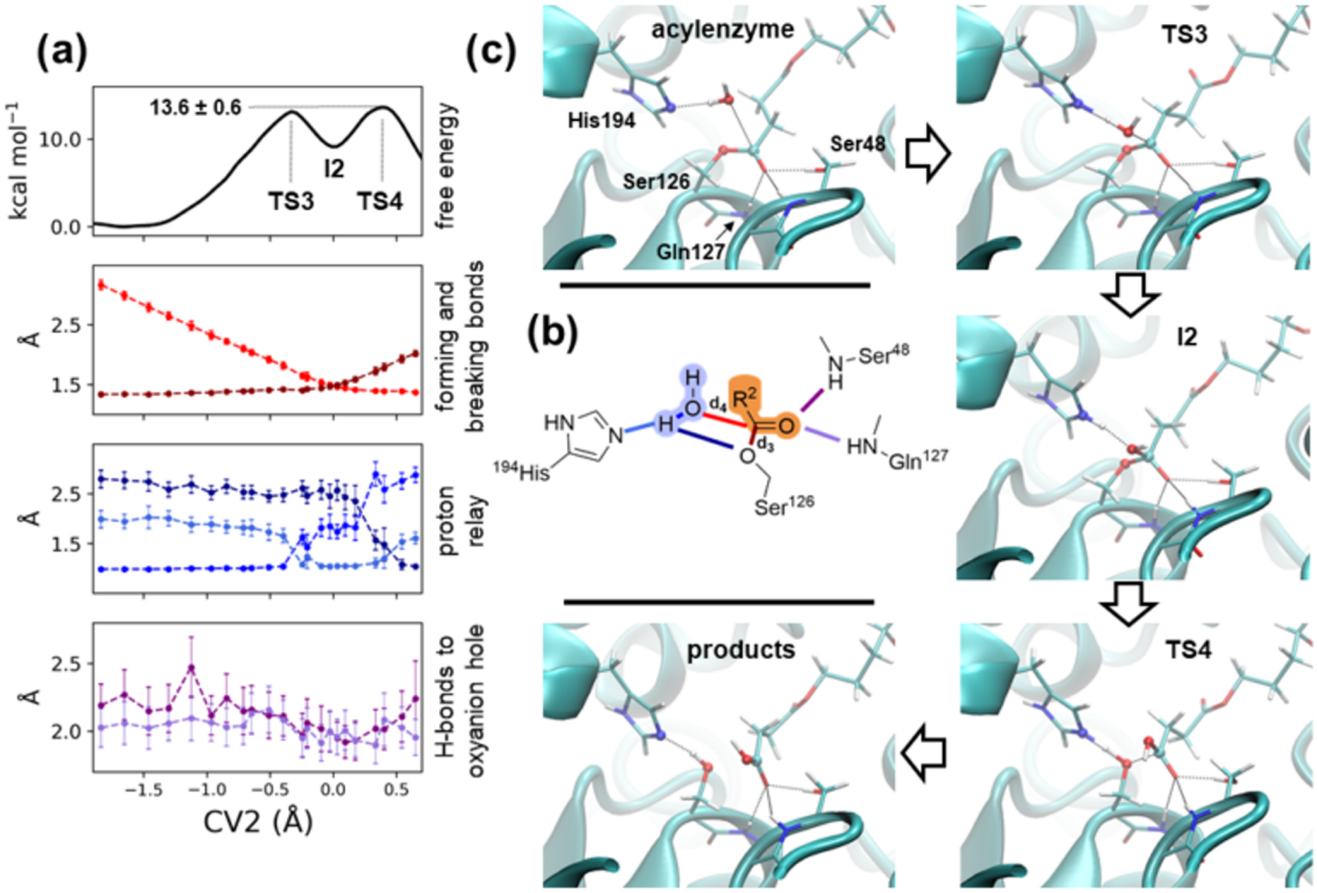
Decylation reaction step. (a) Free energy profile and relevant distances along the collective variable CV2 = *d*_3_ – *d*_4_; (b) chemical drawing highlighting the distances shown in panel (a); (c) representative configurations of reactants, transition states, and intermediate and product states, labeled according to their corresponding CV2 values.

Umbrella sampling QM/MM simulations were performed to reconstruct the free energy profile for the release of the product R^2^C_Suc_O_2_H along a collective variable (CV2) akin to the one used for the acylation step (Figure 5b). Simulations were started from a configuration in which the PBS-like chain occupies the “*upper*” side of the cleft, which allows a water molecule to consistently hydrogen bond to His194 (Figure 4c). Such water molecule is in close proximity to C_Suc_ and can perform a nucleophilic attack on C_Suc_, with His194 relaying a proton from the nucleophilic water to the side chain oxygen O_Ser_ of Ser126. The PBS fragment is released as a carboxylic acid. The free energy profile (Figure 5a) presents a similar shape to the acylation reaction, with two transition states (**TS3** at CV2 = – 0.3 and **TS4** at 0.4 Å). These are close in free energy (13.2 ± 0.5 and 13.6 ± 0.6 kcal mol^–1^, respectively), flanking a metastable intermediate (**I2**) at CV2 = 0.0 Å corresponding to a tetrahedral intermediate (Figure 5c), in which the forming and breaking bonds are of the same length (∼1.5 Å). The proton relay occurs through His194, and it occurs between the two transition states. At TS3, the C_Suc_–O_Ser_ bond is maintained (∼1.4 Å), while the C_Suc_–O_Wat_ distance is ∼1.7 Å. At this point, a proton is transferred from the nucleophilic water to His194, and it remains bound to the histidine until the system reaches **TS4**. At this point, a shift in the H-bonding partner allows the delivery of the proton to Ser126, favoring the lengthening of the C_Suc_–O_Ser_ bond (∼1.8 Å). Notably, the free energy barrier of the deacylation step is lower than that of the acylation step by ∼6 kcal mol^–1^. This is likely due to the interactions of the ester group with the oxyanion hole: the backbone −NH– group of Ser48 and Gln127 interacts with the carboxylic oxygen throughout the reaction (Figure 5a,c fourth panel; in section 9 of the Supporting Information, we report, for comparison, the free energy profile computed from an alternative acyl-enzyme configuration), while these H-bonds form as the reaction proceeded during the acylation step. Interestingly, such H-bond interactions become shorter in the transition state region (−0.4 < CV2 < 0.4 Å). Furthermore, O_Suc_ maintains an H-bond to the side chain hydroxyl of Ser48. At CV2 > 0.55 Å, we released the restraint and observed the evolution of the systems (Figure S16), which restored the enzyme and released the PBS fragment R^2^C_Suc_O_2_H.

## Discussion

Molecular dynamics simulations, based on both molecular mechanical (MM) and hybrid quantum mechanical (QM)/MM potentials, were used to investigate in atomic detail the mechanism of hydrolysis of a polybutylene succinate (PBS) oligomer, namely, a butanediol–succinate–butanediol sequence capped at the termini with acetyl groups (compound **1** in Figure 2). This reaction is catalyzed by the cutinase enzyme from *A. oryzae* (*Ao*Cut). To the best of our knowledge, this is the first mechanistic study addressing the hydrolysis of an aliphatic polyester by a fungal cutinase.

Enzymatic reactions are characterized by the recognition of the substrate, which binds at the catalytic site, and the subsequent chemical reaction, which transforms the substrate into products.^55^ To investigate this catalytic process, we initially used dynamic docking to explore the binding of the smaller compound **2** to the active site of *Ao*Cut.^56^ Then, we repeatedly (10 times) grew compound **1** from **2***in situ* using the alchemically enhanced sampling (ACES) method. These computations were followed by equilibrium MD simulations (each 250 ns long for a cumulative 2.5 μs simulation time) to relax the ligand-enzyme complex. Conformational analysis of these trajectories revealed that compound **1** fits the extended cleft on the surface of the enzyme, laying at a hydrophobic binding canyon that, however, includes few polar residues, including the catalytic triad at the bottom of the cleft (Figure 2). Simulations show that the flexible PBS-like oligomer adapts to the active site cleft (Figure 2), suggesting that other linear polyesters may fit the cleft. Indeed, AoCut has been reported to fully hydrolyze PBSA,^29^ while PLA, which presents a methyl group protruding from the backbone chain, is only partially processed. The hydrolyzable ester moiety of the PBS-like substrate accommodates at the catalytic triad. Interestingly, here, the carboxylic oxygen of the ester moiety does not approach the backbone NH- groups of the oxyanion hole. Yet, it can form H-bonds to the nearby polar residues, namely, Ser48 and Asn90. The catalytic triad Ser126-His194-Asp181 maintains the expected alignment necessary for activating the nucleophilic hydroxyl, in both apo- and substrate-bound states. In the apo form, *Ao*Cut displays limited fluctuations of the segments comprising residues 87–93 and 186–194, both flanking the catalytic site and composing the outer part of the cleft (Figure S3). The two segments face each other and contact residues Asn90/Ala91 and Leu188, narrowing the cleft (Figures S17b and S18). Indeed, we observed that the PBS-like oligomer does not insert into this part of the groove (Figure S6). Thus, we put forward the idea that the mutant Leu188Ala could grant access to this part of the groove, possibly resulting in a more extended binding of the polyester chain and/or favoring the “*upper*” configuration of the acyl-enzyme adduct, in turn enhancing catalytic efficiency.

Such catalytically competent conformations were used to model the chemical reaction by means of QM/MM MD simulations at the DFT(B3LYP)/6–31G**/AMBERff level of theory. Notably, such calculations were performed with the GPU-enabled electronic structure code QUICK integrated with the MD engine SANDER from the AmberTools23 package.^45,57^ As a result, multiscale QM/MM MD simulations could be performed by using the popular hybrid B3LYP DFT functional. Thanks to the performance of the code,^30^ here we could sample and reconstruct the free energy profiles of both the acylation and deacylation reaction steps and, importantly, further explore the acylation mechanism starting from two different conformations. In our hands, with 77 atoms in the QM region, using the 6–31G(d,p) basis set and the B3LYP functional, this QM/MM implementation performed with competitive timing on high end GPUs (24.6 s/MD-step on a single NVIDIA A100, 39.5 s/MD-step on a NVIDIA V100) and respectable timing on consumer GPU (115.7 s/MD-step on a single NVIDIA GeForce RTX2080). This opens to a broader application of such an efficient code that, notably, incorporates long-range electrostatic corrections, not available in other *ab initio* codes interfaced with SANDER for QM/MM calculations.^30,31^ Furthermore, umbrella sampling simulations were performed using the PLUMED interface to SANDER.^58^ It is worth noting that this procedure enables the application of any enhanced sampling technique in the PLUMED repertoire at the DFT/AMBERff level of theory.

Our results indicate that the acylation step is rate-limiting, with a free energy barrier of 20.2 ± 0.6 kcal mol^–1^, ∼6 kcal mol^–1^ higher than that of the deacylation step (13.6 ± 0.6 kcal mol^–1^). No experimental kinetic data are available to validate the computational estimate. Yet, the free energy barrier aligns with computational studies investigating polyethylene terephthalate hydrolysis by the PETase cutinase at a similar level of theory.^20^ Interestingly, in the most recurrent catalytically competent binding mode of compound **1**, the carboxylic oxygen of the hydrolyzable moiety does not form hydrogen bonds to the oxyanion hole backbone −NH– groups. These interactions are, however, recovered as the reaction proceeds. In this way, such H-bonds contribute to stabilizing the tetrahedral species formed by the nucleophilic attack on the carboxylic carbon. Additional H-bonds are established by the carboxylic oxygen with nearby Ser48, which further stabilizes the tetrahedral species. Interestingly, we explored the energetics of the acylation reaction starting from different configurations, corroborating that the higher the number of H-bonds formed by the carbonylic oxygen in the transition state region, the lower the free energy barrier (Figures 3 and 5 and Supporting Information Section 6). Given the importance of stabilizing the developing negative charge on the carboxylic oxygen at the transition state,^28^ we would argue that the formation of a suboptimal number of H-bonds at the oxyanion hole (possibly due to steric hindrance at the substrate/enzyme interface) would likely generate an increase in the activation barrier. Also, for both the acylation and deacylation steps, the free energy profile shows a metastable tetrahedral intermediate, flanked by transition states that coincide with the proton transfer events (Figures 3 and 5): in **TS1** (acylation) and **TS3** (deacylation), a proton is abstracted by His194 from the nucleophile (Ser126 in acylation and a water molecule in deacylation). In **TS2** and **TS4**, a proton is delivered to the leaving group (the alcohol R^1^O_But_H in acylation and Ser126 in deacylation) by protonated His194 after a switch in the H-bonding partner. Interestingly, during these proton relay events, the H-bond between His194 and Asp181 becomes shorter, and at times, the shared proton is transferred to Asp181 (Figure S10). In the acylation step, **TS1** is higher in energy than **TS2** by 2 kcal mol^–1^ (20.2 ± 0.6 and 18.2 ± 0.7 kcal mol^–1^, respectively). In the deacylation free energy profile, **TS3** and **TS4** are similar in energy (13.2 ± 0.5 and 13.6 ± 0.6 kcal mol^–1^, respectively). It is worth noting that the proton transfer events were not explicitly included in the reaction coordinate, which was approximated using the distances of the nucleophilic and leaving groups from the electrophilic center (as done in ref (20)). These proton transfer events coincide with the location of the transition states (Figure S19), and their contribution is projected and accounted for (within the limitations of the sampled time) in the free energy profiles along CV1 and CV2. To investigate the explicit inclusion of the proton transfer events in the reaction coordinate (see refs (59) and (22−24)), we adopted the adaptive string method at the DFTB3 level of theory, closely following Garcia-Meseguer et al.,^23^ who used this method to study the reaction mechanism of polyester hydrolysis by PETase. These results are described in section 9 of the Supporting Information.

As noted above, to the best of our knowledge, this is the first computational mechanistic study addressing the hydrolysis of an aliphatic polyester by a fungal cutinase. On the contrary, the hydrolysis of PET, an aromatic polyester, by the PETase enzyme from the bacterium *I. sakaiensis* has been the focus of recent mechanistic studies, which we will briefly recall here for comparison. Both *Ao*Cut and PETase display an α/β fold (Figure 1), with *Ao*Cut having a shorter sequence. A comparison of the structures reveals that the groove at the active site of *Ao*Cut is more constricted than that in PETase (Figure S17a). The open and shallower cleft in PETase may be advantageous to bind the bulkier and more rigid PET chain (to our knowledge, the activity of *Ao*Cut toward PET has not been reported). Furthermore, the shape of the cleft differs in the two enzymes, being linear in *Ao*Cut while L-shaped in PETase (Figure S17b). In *Ao*Cut, the residues in contact with the PBS-like oligomer include hydrophobic (Ala47, Leu87, Pro88, Ala91, Val183, Leu188, Val190, and Phe195) and polar residues (Ser48, Asn90, Tyr125, Gln127, Thr156, and the catalytic Ser126 and His194—Figure 2). Compared to PETase, where Trp 159 and Trp185 were proposed to interact with the aromatic rings of PET,^24^ no Trp residues are present at the AoCut cleft.

Fernandes and co-workers^20^ used DFT(PBE)/MM umbrella sampling simulations to model the hydrolysis of a PET dimer. For both acylation and deacylation steps, the tetrahedral species resulting from the nucleophilic attack on the carbonyl carbon corresponds to the transition state of the free energy profile rather than a metastable intermediate as found here. In a related study,^21^ the authors used a similar approach to study the MHETase enzyme, using the B3LYP DFT functional. In this case, the tetrahedral species appeared as a high-energy intermediate. It thus seems that such a tetrahedral species can be differently located on the free energy surface, also depending on the DFT functional. Despite this evidence, the reaction proceeds along the canonical esterase mechanism with an activation barrier of the rate-determining step similar to the one found here (20 kcal mol^–1^ for PETase and 19 kcal mol^–1^ for MHETase). Compared to the present study, for which no structural information was available on the Michaelis complex, both these studies benefited from X-ray structures of the enzymes in complex with substrate 1-(2-hydroxyethyl) 4-methyl terephthalate (as for mutated PETase lacking the catalytic Ser, PDB ID: 5HX3) or a nonhydrolyzable substrate analogue (as for MHETase PDB ID: 6QGA). Therefore, our case also demonstrates that the ACES approach may conveniently be used to couple a flexible substrate (such as compound **1**) to the active site cleft of *Ao*Cut.

Boneta et al.^22^ compared the reactivity PETase and an engineered leaf compost cutinase (LCC-ICCG)^13^ toward a PET dimer (MHET_2_) using AM1/MM umbrella sampling further corrected with M06–2X DFT energy evaluations. The enzymes displayed similar free energy surfaces featuring a tetrahedral intermediate in both acylation and deacylation steps, as found in the present study. Acylation turned out to be rate-limiting, with barriers of 18.9 kcal mol^–1^ for PETase and 21.1 kcal mol^–1^ for LCC-ICCG. They also modeled the reactivity of PETase toward MHET_3_, revealing no dependence of the free energy surface on the length of the polymeric chain. On the other hand, Garcia-Meseguer et al.^23^ compared the reactivity of PETase and FAST-PETase, an engineered variant with enhanced hydrolytic activity.^14^ Using the adaptive string method followed by umbrella sampling simulations at the DFTB3/MM level and a trimer PET substrate model, they rationalized the enhanced activity of FAST-PETase in terms of increased basicity of Asp206 (of the catalytic triad), which in turn modulates the properties of the catalytic His resulting in a lower activation barrier (12.1 kcal mol^–1^ for FAST-PETase compared to 16.5 kcal mol^–1^ for the wild-type PETase in the rate-limiting acylation step). Notably, the atomic rearrangements along the string are consistent with those observed in this study of *Ao*Cut. Finally, Burgin et al.^24^ recently applied transition path sampling at the DFTB3/MM level to investigate the reaction mechanism of a PET dimer by wild-type PETase. The reactive trajectories displayed a moving histidine mechanism for proton relay, as observed in the present study, and highlighted the flexibility of an aromatic tryptophan side chain (Trp185 in *Is*PETase, corresponding to Thr156 in *Ao*Cut) as a key element of catalysis. The free energy profiles were reconstructed *via* umbrella sampling simulation along a reaction coordinate identified through likelihood maximization. Deacylation was predicted as rate-limiting with a barrier of ∼18 kcal mol^–1^. However, the tetrahedral species correspond to transition states on both acylation and deacylation profiles rather than to metastable intermediates as found in our study. Nevertheless, all of these mechanistic studies align with our results for the overall canonical esterase mechanism. The reported activation barriers are in the 12 to 20 kcal mol^–1^ range although computed with different QM methods, ranging from semiempirical approaches (AM1 and DFTB3) to DFT (PBE). The nature of the tetrahedral species resulting from the nucleophilic attack was predicted to be either a metastable intermediate or a transition state of the acylation or deacylation steps. This discrepancy is possibly related to the QM level of theory. However, the size of the QM region and the definition of the reaction coordinate may play a role in modulating the stability of such a chemical species. Besides the mechanistic details, which may (slightly) differ due to, for example, a diverse enzyme/substrate combination and distinct methodologies, these studies, together with the present one, provide a consistent picture of the well-established esterase mechanism used for the hydrolysis of non-natural polyesters by cutinases. When the ester group can approach the catalytic triad, the reaction proceeds with activation barriers that align with those expected for enzymatic catalysis. This may constitute a guiding principle for future engineering studies aimed at improving the hydrolysis of polyesters other than PET and PBS.

Using the popular hybrid B3LYP functional, we thus obtained barriers consistent with previous works (20.2 ± 0.6 kcal mol^–1^ for acylation and 13.6 ± 0.6 kcal mol^–1^ for deacylation), with the tetrahedral species corresponding to a metastable intermediate for both acylation and deacylation. We compared the free energy profiles from umbrella sampling simulations of the acylation and deacylation steps performed at the B3LYP/AMBER and DFTB3/AMBER levels of theory (Figure S9). As far as it concerns the hydrolysis of a PBS-like oligomer by *Ao*Cut, both methods predict a tetrahedral intermediate, albeit the free energy barrier is considerably lower at the DFTB3 level (11.8 ± 0.5 kcal mol^–1^). Also, in contrast to earlier studies,^20−24^ we found that the PBS-like oligomer binds to the active cleft without immediately inserting the carbonylic oxygen into the oxyanion hole. Nevertheless, precise interactions are established as the reaction proceeds. These interactions were then further enriched by an H-bond interaction with Ser48. The additional H-bond interaction formed by Ser48 lowers the activation barrier (as demonstrated by computing the free energy profiles in the presence and absence of this interaction), suggesting that mutant Ser48Ala may be less efficient than the wild-type enzyme. It is worth noting that no H-bonding interactions other than those from the oxyanion hole were reported for the carbonylic oxygen of the ester moiety in PETase in the aforementioned studies.^20,22−24^

The present study, and similarly the mechanistic studies on PETase,^20,22−24^ used model substrates composed of a small number of building blocks rather than the actual polyester. This approach facilitates the modeling and provides insight into how a segment of the polymer chain would fit the active site cleft of cutinases. However, we emphasize that the actual substrate is a solid polymer. Necessarily, a more extended enzyme–substrate interface forms than the one investigated with model substrates. Because of this, the kinetics of substrate binding cannot be inferred from that of model substrates, such as the one investigated here. Molecular details of how the cutinase approaches the polymer surface have yet to be unravelled. Recent studies revealed that the coexpression of hydrophobins, which adsorb on solid hydrophobic materials, may be involved in the recruitment of hydrolytic enzymes.^60^

Overall, here, we present mechanistic insights for the hydrolysis of an aliphatic polyester by fungal cutinases. Aliphatic polyesters are considered more degradable than aromatic polyesters, such that they are often labeled as biodegradable. Yet, the time required for their environmental degradation is substantial and thus demands efficient and cost-effective degradation approaches. In this regard, carboxylic ester hydrolases (EC 3.1.1) are enzymes evolved to hydrolyze ester bonds, an activity that motivated their adoption by the chemical industry to drive biochemical transformations.^25,61^ The use of regioselective enzymes constitutes a route toward a closed-loop recycling strategy that would help drastically reduce plastic waste accumulation in the environment. Indeed, an intense research effort was placed in recent years in engineering cutinases to improve their catalytic efficiencies and thermostabilities. Successful studies based on the rational design of bacterial cutinases have demonstrated the feasibility of this approach for PET,^13,14^ a recalcitrant aromatic polyester highly used in packaging (*e.g.*, water bottles). Necessarily, this rational approach to tuning the enzyme activity must be extended to the hydrolysis of other types of polyesters. Fungi, in contrast to bacteria, induce essential alteration of the mechanical properties of the plastic by the growth of the fungus between the polymer layers, thus weakening the fabric body.^62,63^ This cracking action constitutes an advantage over bacteria, which adhere only to the plastic surface. *A. oryzae* is a common filamentous fungus used in traditional Japanese fermentation products (sake, shoyu, and miso) and is classified by the FDA as generally recognized as safe. *Ao*Cut has been reported to hydrolyze aliphatic polyesters PBS and PBSA and to a lesser extent PLA.^29^ In this context, we reveal the mechanistic details of both the substrate recognition and chemical transformation of PBS by *Ao*Cut. We clarify the fundamental interactions for catalysis. The same computational strategy could be employed to explore and define the coupling and reactivity of *Ao*Cut to other polyesters, providing valuable insights to guide the rational engineering of enzyme variants for the hydrolysis of polyesters of different compositions.

## Conclusions

The hydrolysis of a polybutylene succinate (PBS) oligomer by a fungal cutinase (*Ao*Cut) has been studied in atomic detail by means of state-of-the-art molecular modeling techniques, including dynamic docking and advanced alchemical transformations. Also, we run extended QM(B3LYP)/MM umbrella sampling simulations to sample potential reaction paths. In this way, we showed that three units of the aliphatic polyester fit the continuous groove across the active site, interacting with hydrophobic side chains. The substrate ester moiety approaches the catalytic residues. Then, it is hydrolyzed according to a canonical esterase mechanism in which the nucleophilic Ser126 is assisted by His194 and Asp181 residues. The free energy barrier of the acylation step is higher (20.2 ± 0.6 kcal mol^–1^) than that of the deacylation step (13.6 ± 0.6 kcal mol^–1^). Alcohol release from the acylezyme intermediate occurs on a fast (ns) time scale, suggesting that the acylation step is rate-limiting. These findings align with the experimentally determined biodegradation activity of *Ao*Cut toward PBS and provide guiding principles to aid the rational design of cutinases with improved activity toward other types of polyesters.

## Data Availability

Configuration files and structural models to reproduce the calculations reported in this study are provided as Supporting Information (file Vidossich_AoCut_PBS_data.tar). Software used in this study include sander, QUICK, and cpptraj from AmberTools23 (available at https://ambermd.org/GetAmber.php#ambertools), pmemd from Amber22 (available at https://ambermd.org/AmberMD.php), PLUMED 2.8.2 (available at https://www.plumed.org/download), and Glide from Schrodinger2021–3 (available at https://www.schrodinger.com/platform/products/glide/).

## Supporting Information Available

Parametrization of PBS-like oligomers and acylated serine residue; classical MD simulations of apo cutinase; models of the cutinase/compound 2 complex; models of the cutinase/compound 1 complex; acylation reaction pathway from configuration 1_A; acylation reaction pathway from configuration 1_B; classical MD simulations of the acyl-enzyme intermediate; deacylation reaction pathway; adaptive string method calculations, and input and parameter files and structural models to generate topology files and model systems, together with configuration files to reproduce the MD simulations. Sample restart files from each MD simulations. Setup files and one sample structure file used for docking (PDF)
