# Supplementary material for: Aliphatic Polyester Recognition and Reactivity at the Active Cleft of a Fungal Cutinase
Source: J Chem Inf Model. 2025 Apr 24;65(9):4662–73. doi: 10.1021/acs.jcim.5c00739 (PMC12076486; doi:10.1021/acs.jcim.5c00739)
Supplement: Supplementary file 1 — ci5c00739_si_001.pdf [file ci5c00739_si_001.pdf]

Supporting Information

Aliphatic polyester recognition and reactivity at the active cleft of a fungal cutinase

Pietro Vidossich,[1]* Madushanka Manathunga,[2] Andreas W. Götz,[3] Kenneth M. Merz, Jr.[2]* and Marco De Vivo[1]*

[1] Laboratory of Molecular Modeling and Drug Discovery, Istituto Italiano di Tecnologia, via Morego 30, 16163 Genoa, Italy

[2] Department of Chemistry and Department of Biochemistry and Molecular Biology, Michigan State University, 578 S. Shaw Lane, East Lansing, Michigan 48824-1322, United States

[3] San Diego Supercomputer Center, University of California, San Diego, 9500 Gilman Drive, La Jolla, California 92093-0505, United States

Corresponding autor
*Pietro Vidossich – Email: pietro.vidossich@iit.it
*Kenneth M. Merz – Email: merz@chemistry.msu.edu
*Marco De Vivo – Email: marco.devivo@iit.it

Contents

1. **Parametrization of PBS-like oligomers and acylated serine residue**
2. **Classical MD simulations of apo cutinase**
3. **Models of the cutinase / compound 2 complex**
4. **Models of the cutinase / compound 1 complex**
5. **Acylation reaction pathway from configuration 1_A**
6. **Acylation reaction pathway from configuration 1_B**
7. **Classical MD simulations of the acylenzyme intermediate**
8. **Deacylation reaction pathway**
9. **Adaptive String Method calculations**

1. **Parametrization of PBS-like oligomers and acylated serine residue**

Force field parameters for compounds **1** and **2** were derived as follows. Bonded and Van der Waals parameters from GAFF2 were adopted. Atomic partial charges were developed according to the RESP methodology.[1] Molecules **2**, **3, 4** and **5** were energy minimized in an extended conformation at the DFT(B3LYP)/6-31G* level, and the electrostatic potential was calculated on a grid at the HF/6-31G* level. Partial charges were computed with pyRESP.[2] During the fitting process, the sum of point charges of atoms belonging to specific moieties of molecules **2**, **3** and **5** (see Figure S1) were constrained as follows:

ACX 0.22 e

OMX -0.22 e

SUC 0.44 e

BUT -0.44 e

ACE 0.0 e

NME 0.0 e

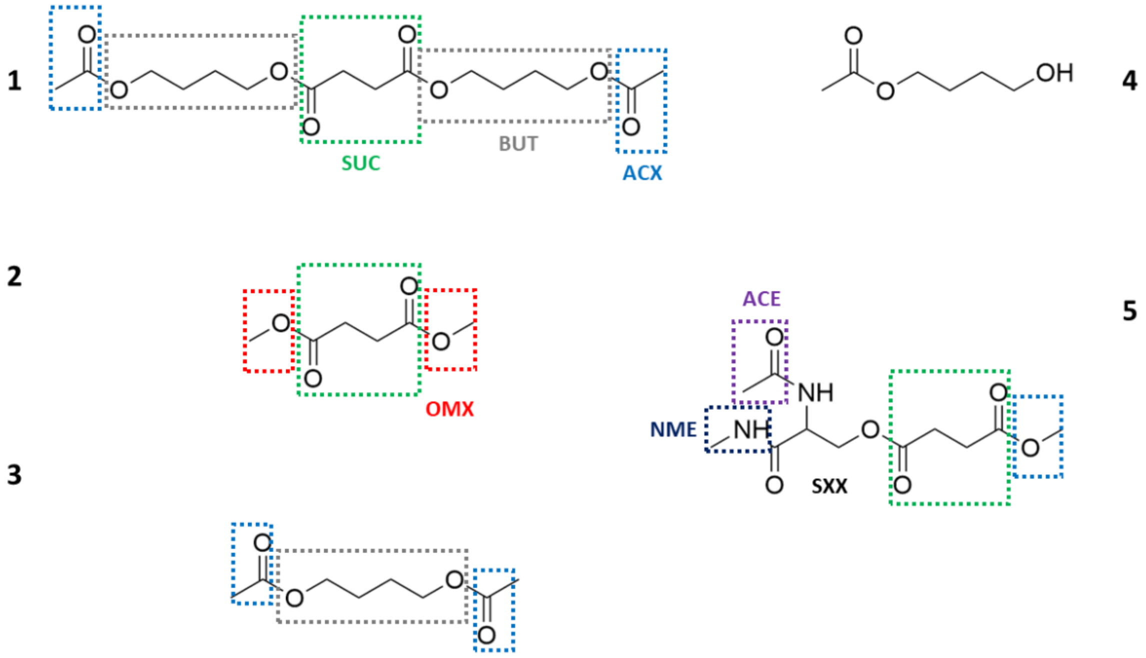

Figure S1. Molecules used to derive atomic charges.

2. **Classical MD simulations of apo cutinase**

Classical 1.1 μ-long MD simulation of the apo enzyme revealed a stiff fold, with only limited fluctuations of the active site cleft (Figure S3). The protein fold is well maintained, with the RMSD of the whole backbone fluctuating around an average value of 1.1 Å, the RMSD of the backbone excluding the first 15 N-terminal residues fluctuating around 0.7 Å, and that of the secondary structure elements fluctuating around 0.5 Å (Figure S2a,c). The profile of the RMSF reflects the protein secondary structure, with smaller values for the Cα atoms of α-helices and β strands (Figure S2b,c). Concerning the catalytic triad (Figure S2d), the H-bond between Asp181 and His194 is maintained throughout the simulation (Figure S2f), while that between Ser126 and His 194 is present in 47% of the trajectory (Figure S2e).

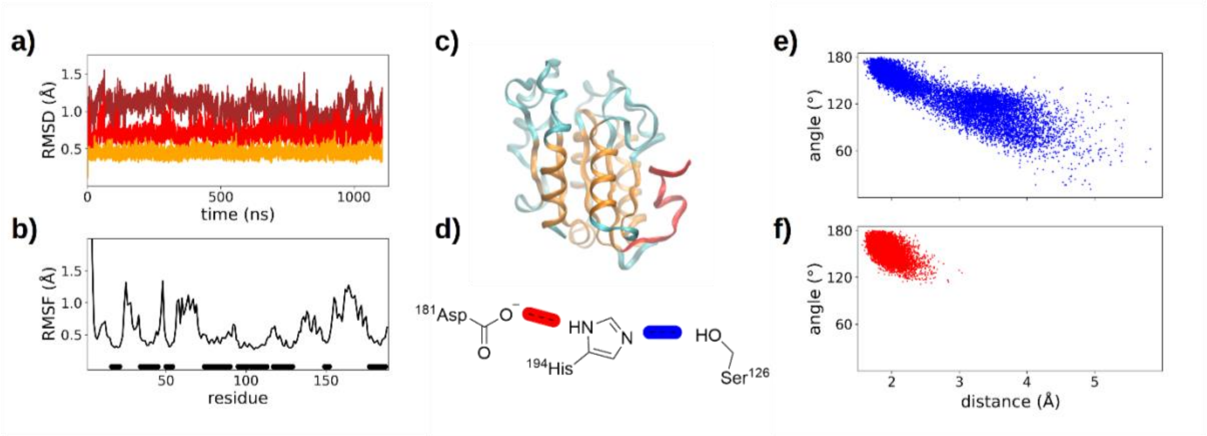

Figure S2. Data from MD simulation of apo *Ao*Cut. a) Root mean squere deviation (RMSD) with respect to the initial structure of the whole protein backbone (brown line), the backbone excluding the first 15 N-terminal residues (red line) and the backbone of the secondary structure elements (orange line); b) root mean square fluctuations of the C_α atoms; on the horizontal axis, the black boxes highlight the residues belonging to elements of secondary structure; c) structure of *Ao*Cut highlighting the first 15 N-terminal residues (red segment) and the residues belonging to secondary structure elements (orange segments); d) H-bond network within the catalytic triad; e) scatter plot of the distance N_ε@His194 – H@Ser126 and angle N_ε@His194 – H@Ser126 – O@Ser126 characterizing the H-bond between Ser126 and His194; f) scatter plot of the distance O@Asp181 – H@His194 and angle O@Asp181 – H@His194 – N_δ@His194 characterizing the H-bond between Asp181 and His194.

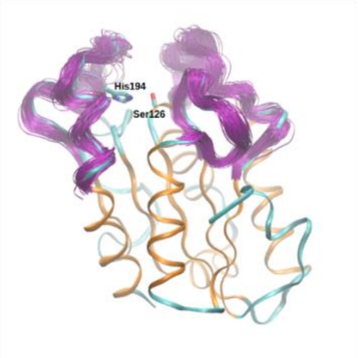

Figure S3. One hundred structures (one every 10 ns) of the protein backbone (purple ribbons) around the catalytic Ser126 and His194 (shown as stick) are superimposed to the initial *Ao*Cut structure to highlight the limited conformational variability of the active site cleft during a 1.1 μ-long MD simulation of apo *Ao*Cut.

3. **Models of the cutinase / compound 2 complex**

We initially investigated the binding of the smaller dimethyl succinate (compound **2**), which comprises the hydrolysable unit of PBS, to the *A.oryzae* cutinase.

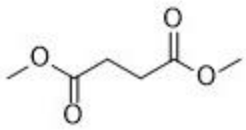

2

Initially, we performed ensemble docking of compound **2** to *Ao*Cut using 1000 structures randomly selected from the MD trajectory of the apo enzyme. The most populated pose consistent with the esterase mechanism was used to start a MD simulation of the *Ao*Cut/**2** complex. We observed that the residence time of compound **2** was short and limited to few ns. Several binding events at the active cleft were observed during a 1 μ-long MD simulation (Figure S4a), which were grouped based on structural similarity. In the most populated cluster consistent with the esterase mechanism (8% of the whole trajectory, conformation **2_A** in Figure S4b), the carboxylic oxygen of compound **2** is forming only one H-bond to the oxyanion hole, while only occasionally the textbook conformation featuring both H-bonds to the oxyanion hole was observed (conformation **2_B** in Figure S4b).

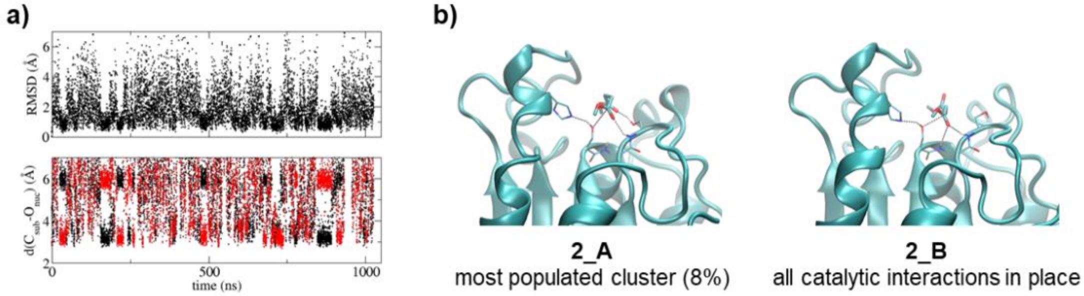

Figure S4. Data from MD simulation of the *Ao*Cut/compound **2** complex. **a)** RMSD with respect to the initial pose (top panel) and distance of either carbonyl carbon of compound **2** from the nucleophilic oxygen of Ser126 (bottom panel); **b)** representative structures of the most populated pose of compound **2** in the active cleft of AoCut (**2_A**) and of the pose expected according to the canonical esterase mechanism (**2_B**).

4. **Models of the cutinase / compound 1 complex**

Based on information retrieved on compound **2**, we then modeled compound **1** in situ. Compound **1** was used as model substrate to study the enzymatic reaction.

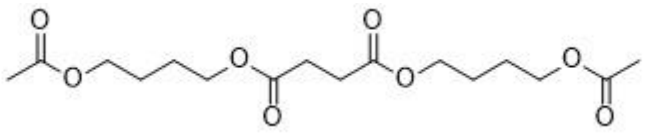

1

Models for the binding of compound **1** to *Ao*Cut were developed from binding pose **2_B** using an alchemical approach. Specifically, we used the alchemical enhanced sampling (ACES) method to "grow" compound **1** from compound **2**, followed by standard MD for a cumulative simulation time of 2.5 μs, which were analyzed to reveal the binding modes of compound **1** to the active cleft of *Ao*Cut. Note that the objective of the alchemical transformation is to couple compound **1** to the active cleft of *Ao*Cut, and not to estimate their affinity.

In all but one of our 10 simulations, compound **1** remained in the active cleft of *Ao*Cut along the 250 ns long MD (Figure S5). The initial conformation of the succinate moiety in all the unbiased simulations was consistent with pose **2_B** of compound **2**, favored by the application of a distance restraint between the carbonyl oxygen of compound **1** and the oxyanion hole H-bond donors during the alchemical transformation. This initial binding conformation was maintained in the initial stages of the simulations (ranging from few ns up to 90 ns) and then rearranged (Figure S5). Thus, compound **1** displayed a longer life-time of the ideal pro-reactive pose compared to compound **2**, likely due to the increased contact surface. In eight simulations, however, alternative binding poses were also visited. We focused the analysis on those displaying a distance < 4 Å between the hydrolysable carbonyl carbon of compound **1** and the nucleophilic hydroxyl group of Ser126, accounting for 64% of the cumulative trajectory. Clustering of these frames based on the RMSD of the central succinate unit revealed a consistent binding mode (50%) in which the carbonyl oxygen is not inserted in the oxyanion hole. A smaller franction (18%) of configurations displayed the carbonyl oxygen H-bonded to the oxyanion hole –NH– groups. Notably, these configurations are found in the initial stages of the MD simulations. We further refined the clustering analysis using the RMSD of the larger butanediol–succinate–butanediol moiety of compound **1**, showing that indeed the substrate had a recurrent (16%) binding mode in the active site cleft of *Ao*Cut (Figure S6), while the acetyl termini of substrate **1** are solvent exposed and display high mobility during the MD simulations. The butanediol–succinate–butanediol moiety accommodates between the side chains of Ala47, Ser48, Leu87, Pro88, Asn90, Ala91, Tyr125, Ser126, Gln127, Thr156, Val183, Leu188, Val190, His194, Phe195. Most interactions are thus hydrophobic, although the polar Ser48, Asn90 and Gln127, in addition to the catalytic Ser126 and His194, surround the hydrolysable ester moiety (see also Figure 2 of the main text).

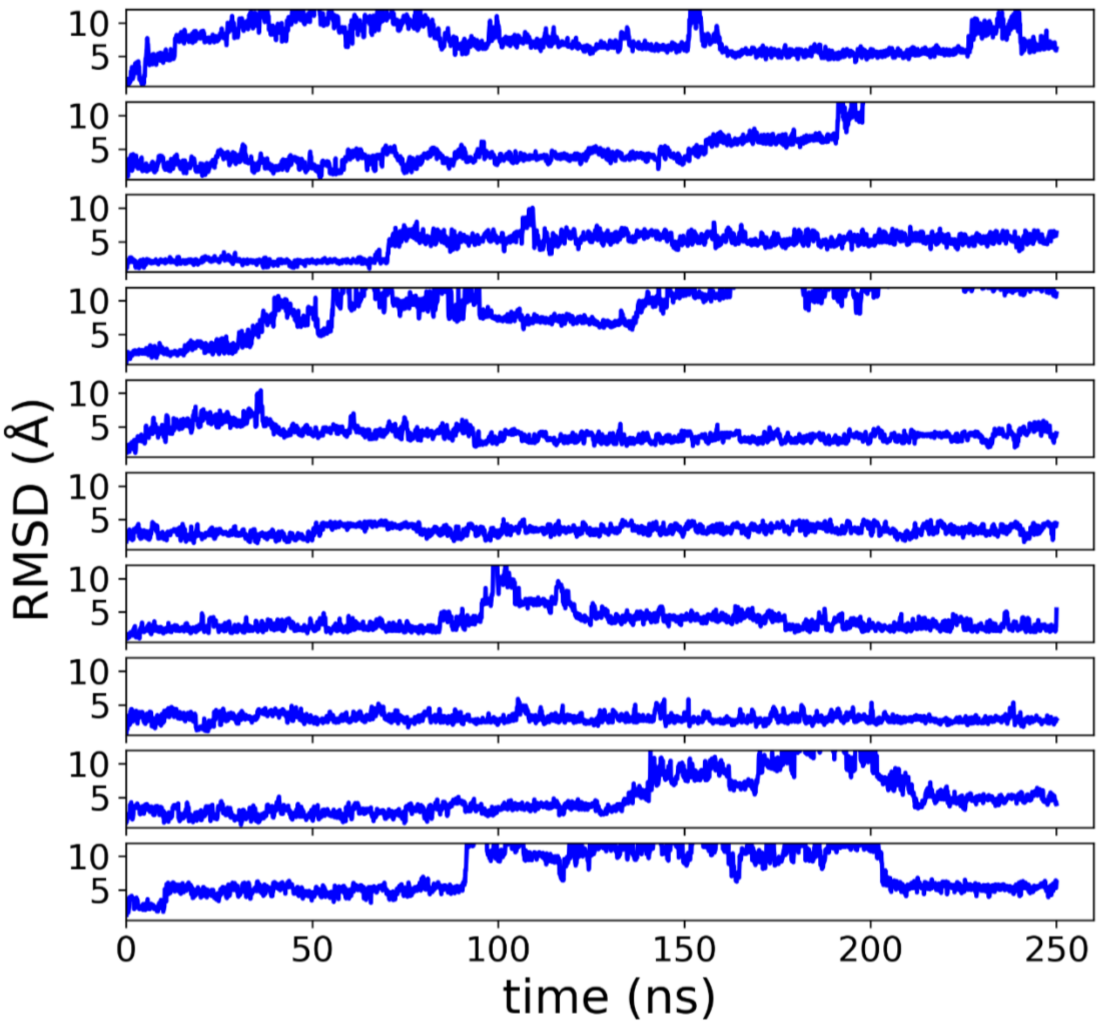

Figure S5. Data from MD simulations of the *Ao*Cut/compound **1** complex. For each of the 10 replicas, the RMSD with respect to the pose at the end of the alchemical transformation are shown (graphs were restricted to the 0 < RMSD < 12 Å range).

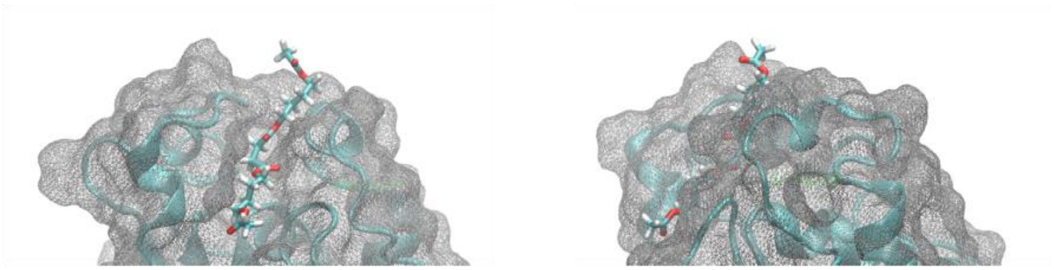

Figure S6. Data from MD simulations of the *Ao*Cut/compound **1** complex. Two views of the most populated pose of compound **1** in the active cleft of *Ao*Cut. The enzyme backbone is rendered as cyan cartoon, while the enzyme surface as grey wireframe.

5. Acylation reaction pathway from configuration 1_A

Table S1. Umbrella sampling simulations. Force constants and centers of the biasing potentials used in each window.

**Window**	**CV1₀** (Å)	**K** (kcal mol^{-1} Å^{-2})
1	-2.25	120
2	-2.05	120
3	-1.85	120
4	-1.65	120
5	-1.45	120
6	-1.25	120
7	-1.05	120
8	-0.85	120
9	-0.65	120
10	-0.65	240
11	-0.65	400
12	-0.6	600
13	-0.55	600
14	-0.45	240
15	-0.35	240
16	-0.25	240
17	-0.15	240
18	-0.05	240
19	0.05	240
20	0.15	240
21	0.25	240
22	0.3	300
23	0.35	240
24	0.45	240
25	0.55	240

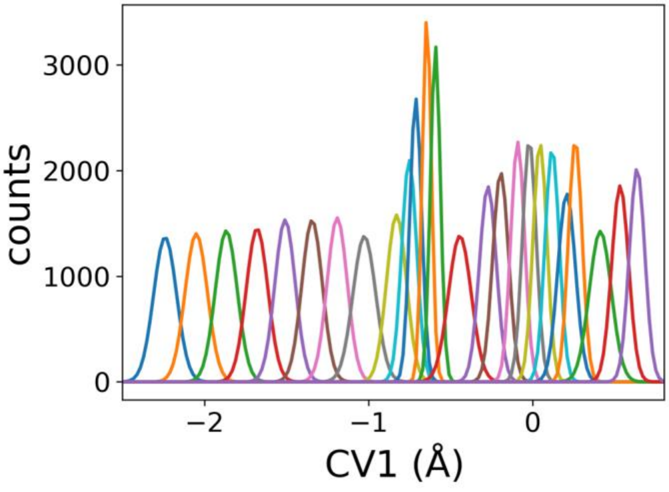

Figure S7. Umbrella sampling simulations. Histograms of the collective variable sampled in each window.

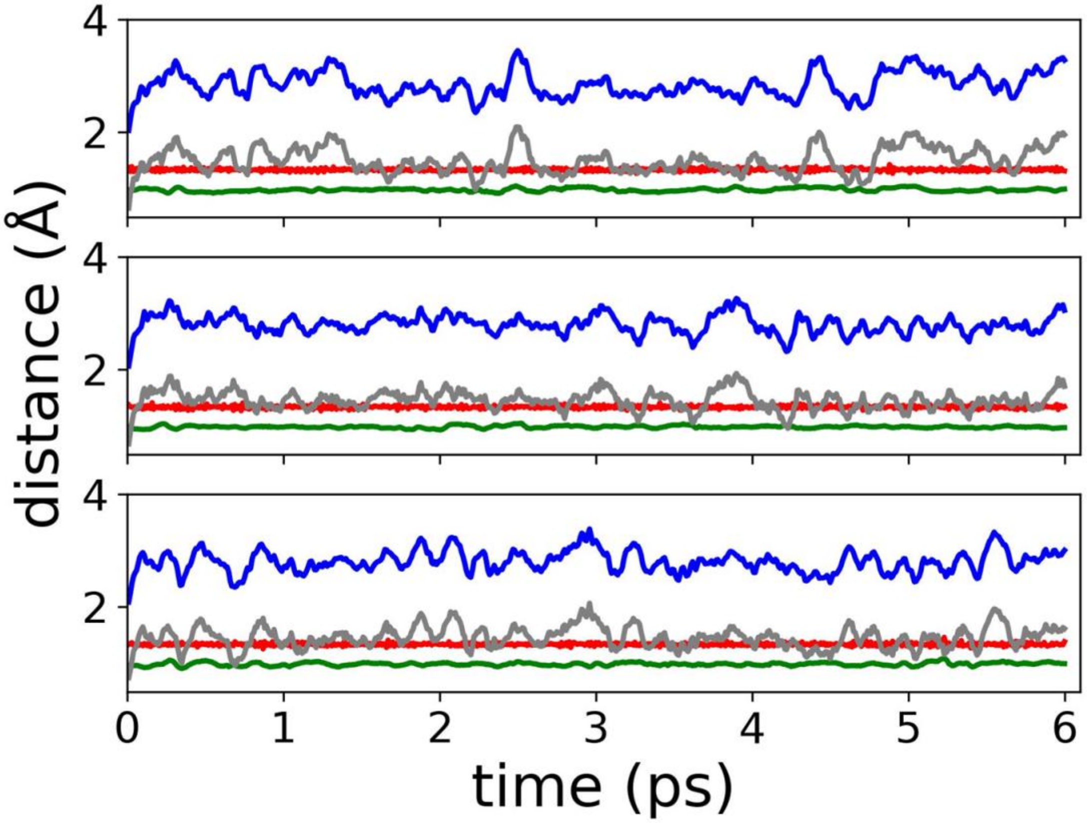

Figure S8. Acylation reaction step from configuration **1_A**. The time evolution of CV1 (grey curve) and of the distances: carboxylic C – leaving O (blue curve), carboxylic C – nucleophilic O (red curve) and leaving O – H from the nucleophile (green curve) are reported along three QM/MM MD simulations started from different configurations belonging to the umbrella sampling window at CV1=0.55 Å. It may be appreciated that CV1 rapidly increases from the initial value of about 0.5 Å and settles around 1.5 Å, which correspond to the lengthening of the carboxylic C – leaving O distance. These simulations confirm that the system rapidly reaches the acylenzyme state, which is stable throughout the rest of the simulations.

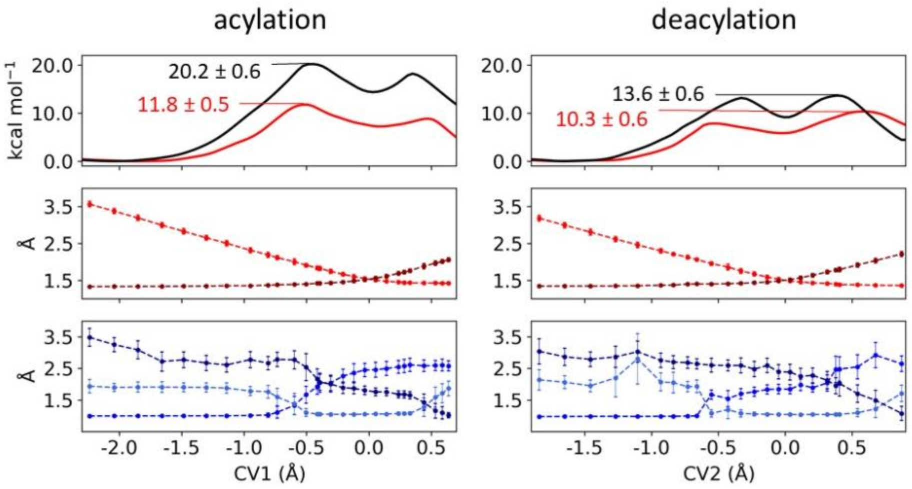

Figure S9. Free energy profiles of acylation and deacylation steps computed using B3LYP/AMBER (black line) and DFTB3/AMBER (red line). Relevant distances are colored as in Figure 3 for acylation and Figure 5 for deacylation steps.

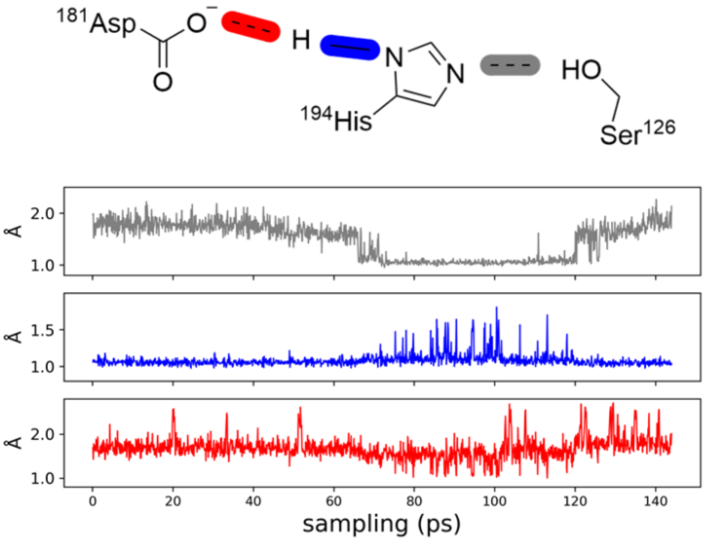

Figure S10. Acylation reaction step from configuration **1_A**. The graphs report the distances highlighted in the scheme during the umbrella sampling simulation, showing how the H-bond between His194 and Asp181 becomes shorter and the proton is at times transferred from His194 to Asp181 when His194 abstracts the proton from Ser126.

6. Acylation reaction pathway from configuration 1_B

Conformation **1_B** represents an alternative binding mode of substrate **1** in the active site cleft of *Ao*Cut. Compared to **1_A**, conformation **1_B** features a similar positioning of the substrate, but displays different interactions at the ester moiety (Figure 3c and Figure S11b). In **1_B**, the carboxylic oxygen is H-bonded to the side chain amide of Asn90 (and not to Ser48 as in **1_A**) and a water molecule trapped between the substrate and the oxyanion hole (Figure S11b). As in **1_A**, the carboxylic oxygen is not H-bonded to the oxyanion hole. QM/MM umbrella sampling simulations were performed to investigate the energetics of the acylation step starting from this configuration, now including the side chain of Asn90 and the water molecule in the QM region. The reconstructed free energy profile from **1_B** displays a metastable intermediate (I1') flanked by two transition states of similar energy (TS1' at 24.0 ± 0.6 kcal mol^{-1} and TS2' at 24.5 ± 0.7 kcal mol^{-1}, Figure S11a). Notably, **1_B** showed a higher barrier than **1_A** (20.2 ± 0.6 kcal mol^{-1}). The atomic rearrangements taking place as the reaction proceeds are similar to those observed for conformation **1_A**, with the moving His194 relying a proton from Ser126 to the leaving group of the substrate. However, in **1_B** the H-bonding interactions with the oxanion hole are not fully established, and only one H-bond to Gln127 is formed (Figure S11a). Furthermore, the water molecule is displaced from its initial binding pose as C_{Suc} of compound **1** approaches the nucleophilic hydroxyl of Ser126, which also leads to the breaking of the H-bond between the substrate and Asn90. As a result, in the transition state region the tetrahedral intermediate only features two H-bonds to O_{Suc}, likely explaining the higher free energy barrier along this pathway.

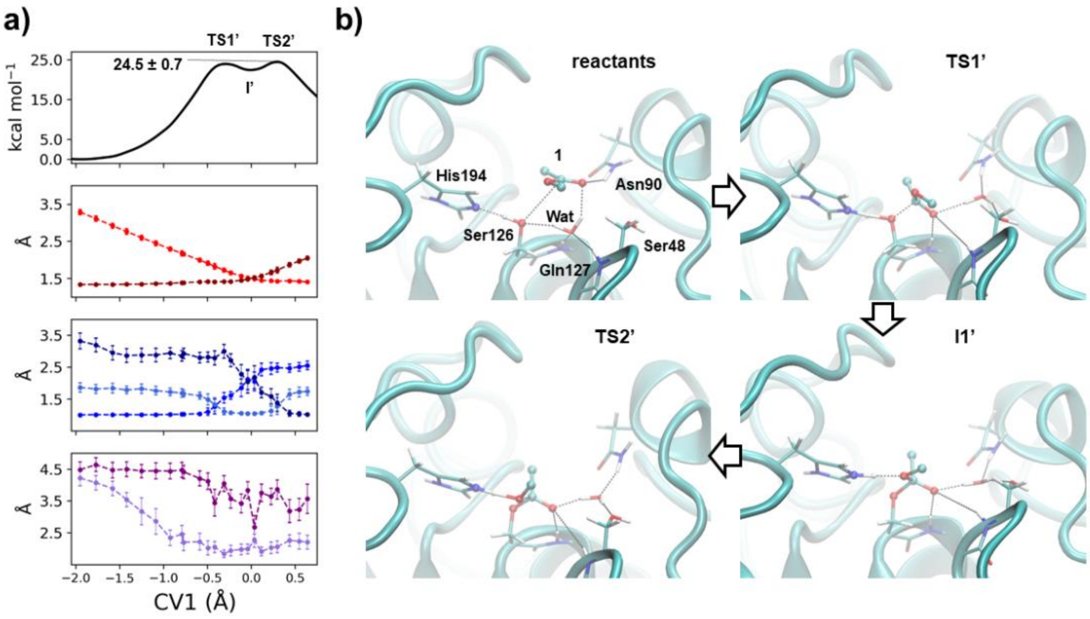

Figure S11. Acylation reaction step from conformation **1_B**. **a)** Free energy profile and relevant distances along the collective variable CV1 (see Figure 3 for CV1 definition); **b)** representative configurations of reactants, transitions states and intermediate. For the sake of clarity, only the hydrolysable moiety of compound **1** is drawn.

Table S2. Umbrella sampling simulations. Force constants and centers of the biasing potentials used in each window.

**Window**	**$CV1_0$** (Å)	**K** (kcal mol^{-1} $Å^{-2}$)
1	-1.95	120
2	-1.75	120
3	-1.55	120
4	-1.35	120
5	-1.15	120
6	-0.95	120
7	-0.75	120
8	-0.55	120
9	-0.65	240
10	-0.45	240
11	-0.4	300
12	-0.35	240
13	-0.3	300
14	-0.25	240
15	-0.15	240
16	-0.05	240
17	0.05	240
18	0.15	240
19	0.25	240
20	0.3	300
21	0.35	240
22	0.45	240
23	0.55	240

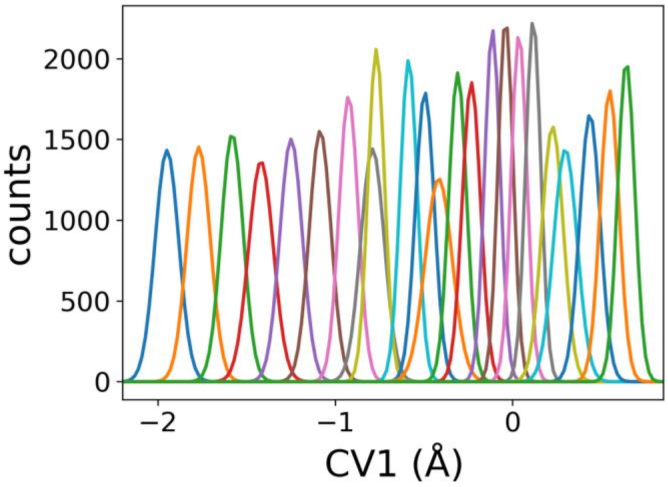

Figure S12. Umbrella sampling simulations. Histograms of the collective variable sampled in each window.

7. Classical MD simulations of the acylenzyme intermediate

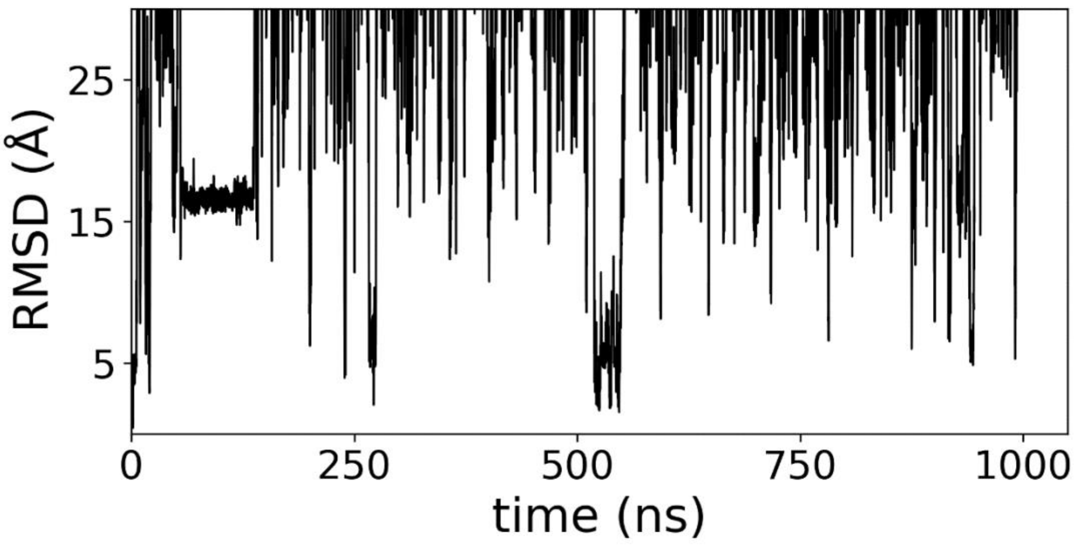

Figure S13. MD simulation of the acylenzyme / $R^1O_{But}H$ complex. Root mean square deviation (RMSD) of the $R^1O_{But}H$ product with respect to its pose at the end of the acylation step. $R^1O_{But}H$ leaves the active site cleft in the early stages of the simulation and only occasionally binds back to the active site.

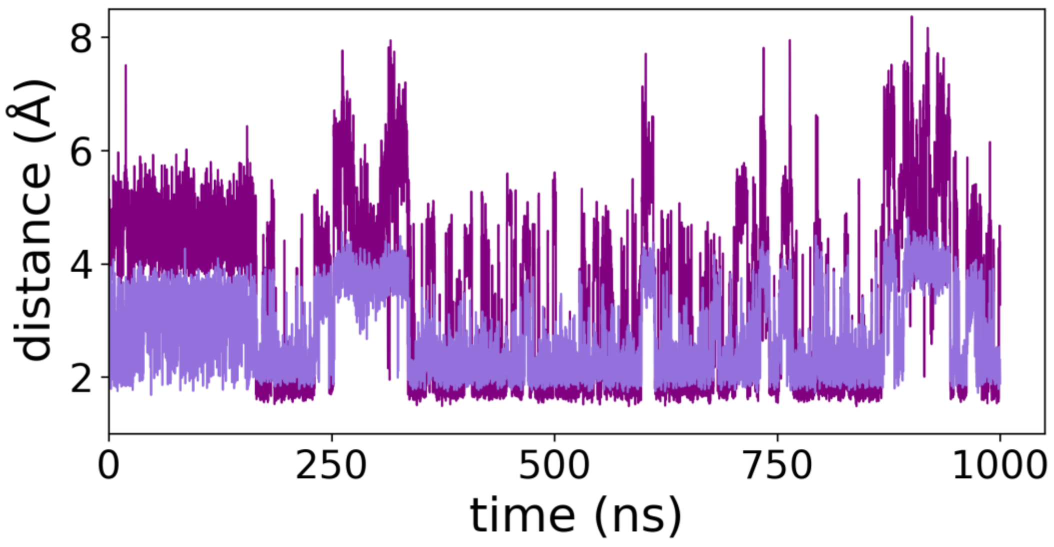

Figure S14 MD simulation of the acylenzyme system. The distances between O_{Suc} and the backbone –NH– hydrogens of the Ser48 and Gln127 are reported. Likely due to the spatial proximity imposed by the covalent bond formed by the substrate with Ser126, O_{Suc} forms H-bond interactions with the oxyanion hole more easily than the unbound compound **1** in the Michaelis complex.

8. **Deacylation reaction pathway**

Table S3. Umbrella sampling simulations. Force constants and centers of the biasing potentials used in each window.

**Window**	**$CV2_0$** (Å)	**K** (kcal mol^{-1} $Å^{-2}$)
1	-1.85	120
2	-1.65	120
3	-1.45	120
4	-1.25	120
5	-1.05	120
6	-0.85	120
7	-0.8	360
8	-0.65	240
9	-0.6	360
10	-0.45	240
11	-0.35	240
12	-0.3	360
13	-0.25	240
14	-0.15	240
15	-0.05	240
16	0.05	240
17	0.15	240
18	0.25	240
19	0.35	240
20	0.4	360
21	0.45	240
22	0.55	240

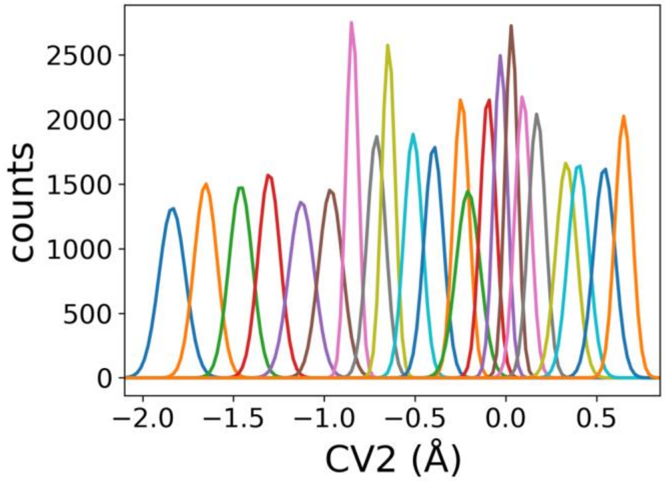

Figure S15. Umbrella sampling simulations. Histograms of the collective variable sampled in each window.

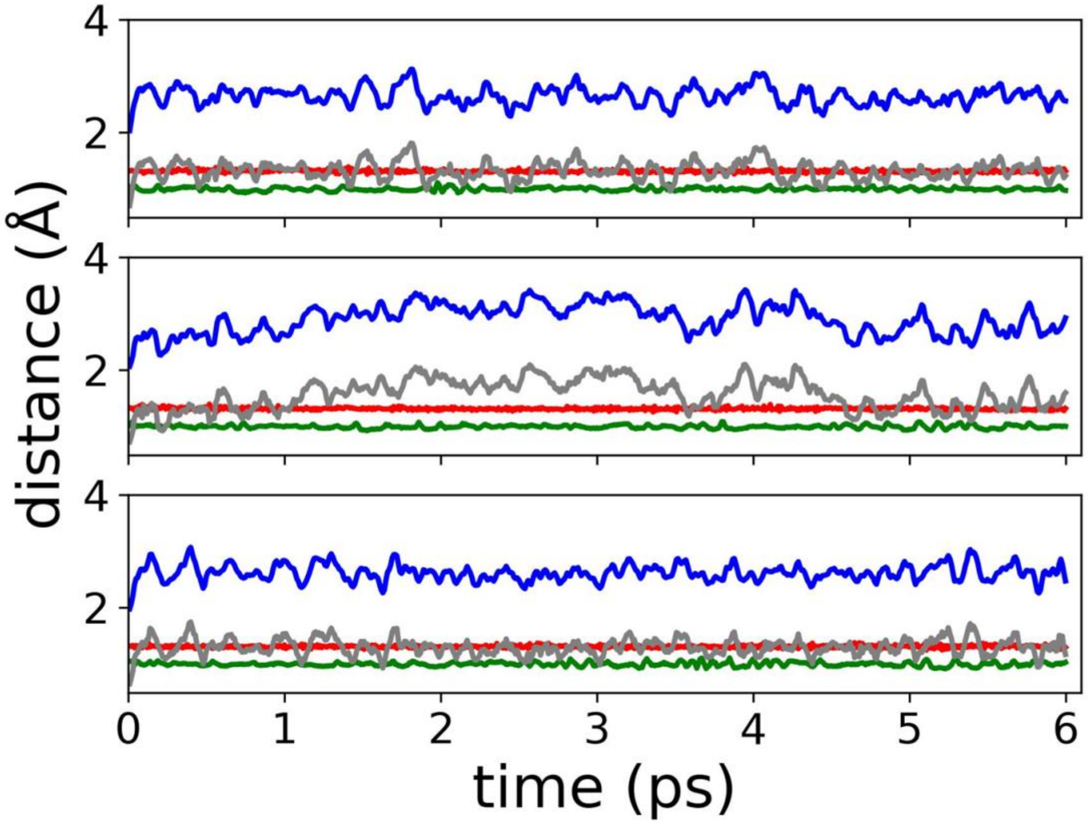

Figure S16. Deacylation reaction step. The time evolution of CV2 (grey curve) and of the distances: carboxylic C – leaving O (blue curve), carboxylic C – nucleophilic O (red curve) and leaving O – H from the nucleophile (green curve) are reported along three QM/MM MD simulations started from different configurations belonging to the umbrella sampling window at CV2=0.55 Å. It may be appreciated that CV2 rapidly increases from the initial value of about 0.55 Å and settles around 1.3 Å, which correspond to the lengthening of the carboxylic C – leaving O distance. These simulations confirm that the system rapidly reaches the product state, which is stable throughout the rest of the simulations.

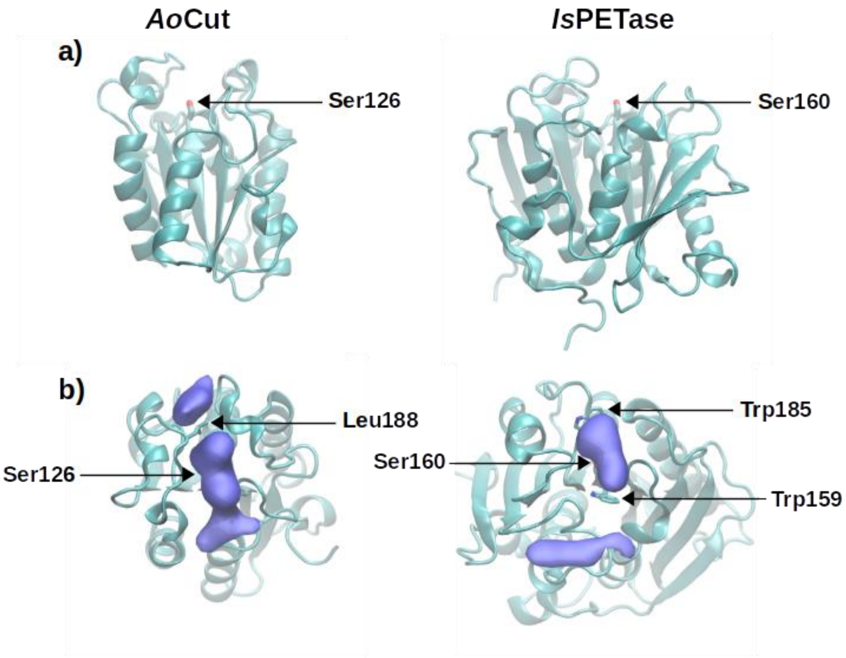

Figure S17. Structures of *Ao*Cut (PDB ID: 3GBS) and *Is*PETase (PDB ID: 6EQE). Views from the side (a) and from the top (b) of the active site cleft. In panel b), the solid surfaces enclose the accessible volumes as computed with fpocket.[3]

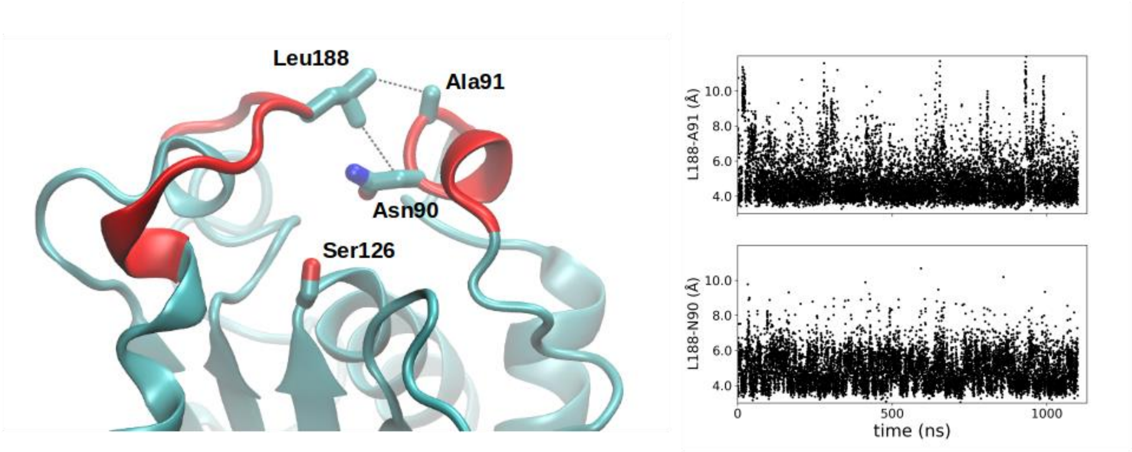

Figure S18. MD simulation of apo *Ao*Cut. The interactions between Leu188 and Asn90/Ala91 narrow the active site cleft. These interactions are maintained throughout the simulation. Segments 186–194 and 87–93 are colored in red.

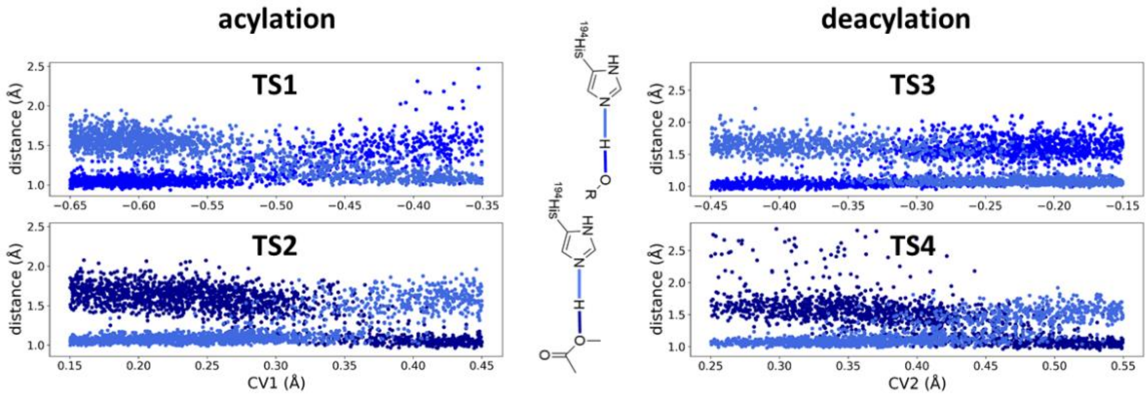

Figure S19. Distances of the transferring proton from donor and acceptor atoms along the collective variables CV1 (acylation) and CV2 (deacylation) in the regions corresponding to the transition states. As may be appreciated, several proton transfer events take place around CV1=-0.5 (TS1), CV1=0.3 (TS2), CV2=-0.3 (TS3) and CV2=0.4 (TS4).

9. **Adaptive String Method calculations**

Nucleophilic attack on the carbonyl carbon during both acylation and deacylation steps is accompanied by a proton transfer from the nucleophile (either Ser126 or a water molecule) to the leaving group through a relay mechanism mediated by His194. In order to investigate to which extent this proton transfer contributes to the free energy profile, we adopted the adaptive string method at the DFTB3 level of theory,[4] closely following Garcia-Meseguer et al.,[5] who used this method to study the reaction mechanism of polyester hydrolyisis by PETase. Accordingly, the minimum free energy path was located in the space defined by the following distances: O_{Ser}–C_{Suc}, C_{Suc}–O_{But}, O_{Ser}–H_{Ser}, H_{Ser}-N_{His}, H_{Ser}–O_{But} for acylation (Scheme 1) and O_{Wat}–C_{Suc}, C_{Suc}–O_{Ser}, O_{Wat}–H_{Wat}, H_{Wat}-N_{His}, H_{Wat}–O_{But} for deacylation. The strings were represented by 64 nodes. The 64 replicas were initially distributed along a path extracted from the umbrella sampling simulations along CV1 (acylation) and CV2 (deacylation). The string was relaxed until stabilization (requiring about 50 ps). 15 ps after string settlement were averaged to define a path CV,[6] which was then sampled via umbrella sampling simulations (15 ps sampling per window were performed). Given the computational cost of relaxing the string to find the minimum free energy path, these calculations were performed at the DFTB3 level of theory.

Results from the string method are in line with the umbrella sampling simulations along CV1 and CV2, which did not include explicitly any proton transfer variable. For both steps, the free energy profile along the minimum free energy pathway presents two transition states separated by a metastable tetrahedral intermediate, in which the forming and breaking bonds are of similar length (Figure S20). The acylation step is rate limiting, with an activation barrier of 12.0 ± 0.5 kcal mol^{-1} (compared to 11.8 ± 0.5 kcal mol^{-1} along CV1 at the at the same level of theory, Figure S9), while the barrier is 7.8 ± 0.5 kcal mol^{-1} for deacylation (compared to 10.3 ± 0.6 kcal mol^{-1} along CV2, Figure S9). The first proton transfer (from nucleophile to His194) anticipates the corresponding transition state, while the proton transfer from His194 to the leaving group occurs in correspondence with the second transition state. This feature, however, appears not to be due to the richer description of the reaction coordinate, but rather to the DFTB3 energy function, as it occurs also in the umbrella sampling simulations along CV1 and CV2 (Figure S9, showing the H-transfers occurring at CV1 = CV2 = –0.6 Å while the transition states are at CV1 = CV2 = –0.5 Å). On the contrary, at the B3LYP level, the proton transfer events were associated with the transition states (Figure S19).

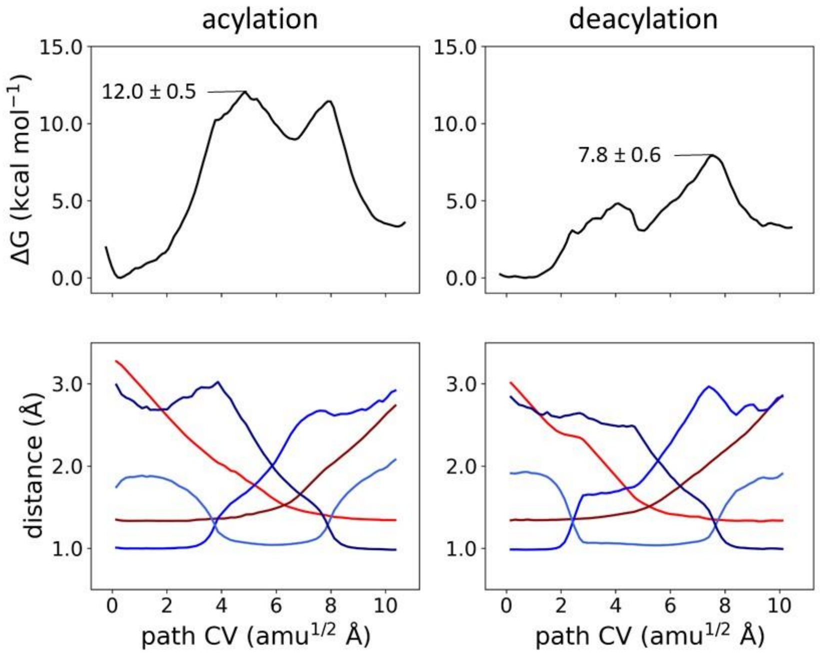

Figure S20. Adaptive string calculations of acylation and deacylation steps. *Top*: free energy profiles. *Bottom*: evolution of the distances involved in the nucleophilic attack and proton transfer relay mechanism, which were used to define and search the minimum free energy paths. Distances are color coded as in Figures 3b (acylation) and 5b (deacylation).

A further adaptive string calculation was performed for the deacylation step, starting from a configuration in which O_{Suc} was not inserted in the oxyanion hole, thus not hydrogen bonded to Ser48 and Gln127. To this aim, we scanned the 1 μs-long classical MD simulation of the acyl-enzyme intermediate in search for configurations in which the distance between the O_{Suc} and both the backbone and hydroxyl hydrogens of Ser48 and Gln127 was larger than 4 Å. These configurations constitute only a minor fraction of the trajectory (3%). Among these, we selected one in which the polyester chain had a similar orientation as that used to investigate the deacylation step starting from O_{Suc} inserted in the oxyanion hole (Figure 5 and S20) and a water molecule hydrogen bound to His194. A steered MD simulation was performed to provide initial configurations to initialize the adaptive string calculation. The string, represented by 64 replicas, was defined in the space of eight distances: O_{Wat}–C_{Suc}, C_{Suc}–O_{Ser126}, O_{Wat}–H_{Wat}, H_{Wat}-N_{His194}, H_{Wat}–O_{But} (as used for the deacylation step shown in Figure S20) plus O_{Suc}–HN_{Ser48}, O_{Suc}–HO_{Ser48}, O_{Suc}–HN_{Gln127}, defining the hydrogen bonds at the oxyanion hole. The corresponding free energy profile is shown in Figure S21, together with the evolution of relevant distance along the string. The profile is consistent with that in Figure S20 (which started from a configuration featuring O_{Suc} inserted in the oxyanion hole), displaying two transition states separated by a tetrahedral intermediate characterized by equal distances between C_{Suc} and the attacking and leaving oxygens. The proton transfer follows the expected relay mechanism through His194 (again, the first proton

transfer to His194 anticipates the first transition state). The hydrogen bonds between O_{Suc} and the oxyanion hole form progressively, starting with the backbone –NH– of Gln127, and are fully formed just before the first transition state. Notably, the activation barrier (13.7 ± 0.9 kcal mol^{-1}) is higher than the one observed for the deacylation step starting from a configuration featuring O_{Suc} inserted in the oxyanion hole (7.8 ± 0.5 kcal mol^{-1}, Figure S20).

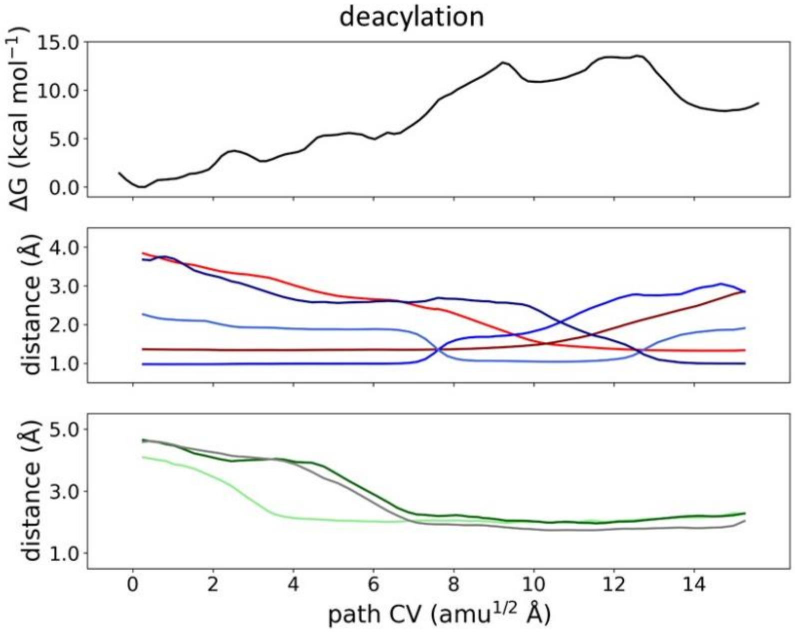

Figure S21. Adaptive string calculation of the deacylation step starting from an alternative configuration, in which the carbonyl oxygen is not inserted in the oxyanion hole. *Top*: free energy profile. *Middle*: evolution of the distances involved in the nucleophilic attack and proton transfer relay mechanism (color coded as in Figures 5b and Figure S20). *Bottom*: evolution of the distances involved in the formation of hydrogen bonds between the carboxylic oxygen and the oxyanion hole, namely O_{Suc}–HN_{Ser48} (dark green), O_{Suc}–HO_{Ser48} (grey), O_{Suc}–HN_{Gln127} (light green). All eight distances were used to define the minimum free energy path traced by the string.

References

1. Bayly, C. I.; Cieplak, P.; Cornell, W.; Kollman, P. A., A well-behaved electrostatic potential based method using charge restraints for deriving atomic charges: the RESP model. *The Journal of Physical Chemistry* **1993,** *97* (40), 10269-10280.
2. Zhao, S.; Wei, H.; Cieplak, P.; Duan, Y.; Luo, R., PyRESP: A Program for Electrostatic Parameterizations of Additive and Induced Dipole Polarizable Force Fields. *Journal of chemical theory and computation* **2022,** *18* (6), 3654-3670.
3. Le Guilloux, V.; Schmidtke, P.; Tuffery, P., Fpocket: An open source platform for ligand pocket detection. *BMC Bioinformatics* **2009,** *10* (1), 168.
4. Zinovjev, K.; Tuñón, I., Adaptive Finite Temperature String Method in Collective Variables. *The Journal of Physical Chemistry A* **2017,** *121* (51), 9764-9772.
5. García-Meseguer, R.; Ortí, E.; Tuñón, I.; Ruiz-Pernía, J. J.; Aragó, J., Insights into the Enhancement of the Poly(ethylene terephthalate) Degradation by FAST-PETase from Computational Modeling. *Journal of the American Chemical Society* **2023,** *145* (35), 19243-19255.
6. Branduardi, D.; Gervasio, F. L.; Parrinello, M., From A to B in free energy space. *The Journal of Chemical Physics* **2007,** *126* (5).
